# *SMO* mutation predicts the effect of immune checkpoint inhibitor: From NSCLC to multiple cancers

**DOI:** 10.3389/fimmu.2022.955800

**Published:** 2022-11-03

**Authors:** Wenxiang Ji, Xiaomin Niu, Yongfeng Yu, Ziming Li, LinPing Gu, Shun Lu

**Affiliations:** Shanghai Lung Cancer Center, Shanghai Chest Hospital, School of Medicine, Shanghai Jiao Tong University, Shanghai, China

**Keywords:** *SMO*, immune checkpoint inhibitor, pan-cancers, tumor immune microenvironment (TIME), biomarker

## Abstract

**Background:**

The emergence of immune checkpoint inhibitors (ICIs) is one of the most promising breakthroughs for the treatment of multiple cancer types, but responses vary. Growing evidence points to a link between developmental signaling pathway-related genes and antitumor immunity, but the association between the genomic alterations in these genes and the response to ICIs still needs to be elucidated.

**Methods:**

Clinical data and sequencing data from published studies and our cohort were collected to analyze the association of the mutation status of *SMO* with the efficacy of ICI therapy in the non-small cell lung cancer (NSCLC) cohort and the pan-cancer cohort. Furthermore, the correlation between *SMO* mutation and immunotherapeutic biomarkers such as immune cell infiltration, immune-related genes, and underlying signaling pathways was analyzed. Three *SMO* mutant plasmids were transfected into cells to explore the *SMO* mutation status in the context of its expression and cell growth.

**Result:**

In the NSCLC discovery cohort, the median progression-free survival in the *SMO* mutant (SMO_MUT) was longer than that in the wild type (SMO_WT) (23.0 vs. 3.8 months, adjusted *p* = 0.041). This finding was further confirmed in the NSCLC validation cohort (8.7 vs. 5.1 months, adjusted *p* = 0.013). In the pan-cancer cohort (*n* = 1,347), a significant overall survival advantage was observed in patients with *SMO* mutations [not reached (NR) vs. 18 months, adjusted *p* = 0.024]. In the subgroup analysis, the survival advantage of SMO_MUT against SMO_WT was prominent and consistent across genders, ages, treatment types, cancer types, and the tumor mutation burden (TMB) status (all *p*_interaction_ > 0.05). In an *in vitro* experiment, we found that both the mutant and wild-type plasmids can promote the expression of *SMO*, but the mutant plasmid had lower *SMO* mRNA and protein levels than the wild type. In CCK-8 experiments, we found that SMO_MUT plasmids can improve the growth of Calu-1 and PC-9 cells, but this capability varied between different mutations and cells. Upon further exploration, the *SMO* mutation status was found to be related to a higher TMB, more neoantigen load, more DNA damage repair (DDR) mutations, higher microsatellite instability (MSI) score, and higher CD8^+^ T-cell infiltration.

**Conclusions:**

The *SMO* mutation status is an independent prognostic factor that can be used to predict better clinical outcomes of ICI treatment across multiple cancer types.

## Introduction

Immune checkpoint inhibitors (ICIs) targeting programmed cell death 1/programmed cell death ligand 1 (PD-1/PD-L1) and/or cytotoxic T lymphocyte antigen-4 (CTLA-4) have made impressive breakthroughs in the treatment of multiple cancer types, ranging from non-small cell lung cancer (NSCLC) to various solid malignant tumors (1–3). However, the clinical response varies among patients, and only a subset of patients can gain long-lasting clinical benefits from ICIs (4). Thus, more precise biomarkers are urgently needed to make ICI treatment more reasonable and to improve its cost-effectiveness.

On account of the complexity of the tumor immune microenvironment (TIME), the factors affecting the clinical effects of ICIs are multifaceted, including PD-L1 expression, tumor mutation burden (TMB), high microsatellite instability (MSI-H), gene expression profiles (GEPs), specific gene mutations, and several specific gut microbiomes (2, 5–9). However, even the commonly used markers, such as PD-L1 expression and TMB, still have limitations in clinical practice (10, 11). Therefore, it is urgent to continue the exploration of novel predictive biomarkers in order to maximize the clinical benefits of ICIs.

Cancer stem cells (CSCs) with intrinsic self-renewal characteristics often play important roles in tumorigenesis, cancer relapse, and metastasis. Developmental signaling pathways that control cell renewal and differentiation are usually reactivated in CSCs, including the Notch, Wnt, and Hedgehog pathways. The interactions of CSCs with the TIME protect the CSCs from being attacked by the immune system (12). Activation of the Wnt/β-catenin signaling pathway may impair dendritic cell recruitment and lead to immune cell exclusion in melanoma and colorectal cancer (13, 14). Activation of the Notch pathway and recruitment of tumor-associated macrophages (TAMs) in breast cancer induce the immunosuppressive microenvironment (15). In this study, we aimed to explore the relationship between the mutation status of the developmental signaling pathway-related genes (including the Notch, Wnt, and Hedgehog pathways) and the treatment efficacy of ICIs.

After a comprehensive analysis of the candidate genes, we found that *SMO* mutation is an independent prognostic factor that can predict better clinical outcomes in patients receiving ICI therapy not only in NSCLC but also in other types of solid cancers. We further uncovered that tumors with *SMO* mutations were more likely to be associated with increased tumor immunogenicity, enhanced antitumor immune microenvironment, and activated antitumor immune signaling pathways.

## Materials and methods

### NSCLC clinical cohort

We collected the developmental signaling pathway-related genes from the Kyoto Encyclopedia of Genes and Genomes (KEGG) database to screen for candidate gene mutations that are potentially related to the efficiency of ICIs (**Supplementary Table S1**). We also collected the data of 349 patients with NSCLC who received ICI treatment from three previously published studies on cBioPortal (https://www.cbioportal.org) and included these as the NSCLC discovery cohort (16–18). Among them, the samples from Rizvi et al. were detected with MSK-IMPACT (Integrated Mutation Profiling of Actionable Cancer Targets) sequencing (*n* = 240). A total of 314 stage IIIB/IV NSCLC patients receiving ICI treatment in Shanghai Chest Hospital between January 2018 and May 2021 were retrospectively analyzed as the independent NSCLC validation cohort, with the follow-up data collection terminating on December 31, 2021. The *SMO* mutation status was identified using next-generation sequencing (NGS) according to the Lung Core Panel with 68 lung cancer-related genes (**Supplementary Table S2**). The detailed sequencing procedure was described in our previous study (19). Data processing is described in **Supplementary Figure S1A**.

### Pan-cancer clinical cohort

Samples from Samstein et al. (*n* = 1347) were included for survival analysis to further explore the prognostic value of *SMO* mutation in pan-cancers (6). The Cancer Genome Atlas (TCGA) pan-cancer cohort (*n* = 3,310) and the non-ICI cohort from Zehir et al. (20) (*n* = 5,588) were taken as controls to investigate whether the survival benefit still exists in patients with *SMO* mutations who did not receive ICI therapy. The data treatment scheme is depicted in **Supplementary Figures S1B, C**.

### Evaluation criteria for clinical efficacy

The objective response rate (ORR), durable clinical benefit (DCB), no durable benefit (NDB), progression-free survival (PFS), and overall survival (OS) were the primary clinical outcomes considered for the evaluation of the treatment effect. ORR was assessed according to the Response Evaluation Criteria in Solid Tumors (RECIST) version 1.1, including complete response (CR) and partial response (PR). DCB was defined as CR, PR, or stable disease (SD) lasting longer than 6 months, whereas NDB included progressive disease (PD) or SD lasting less than 6 months (16). PFS was assessed from the date the patient began immunotherapy to the date of progression or death. OS was calculated from the start date of ICI treatment in the Samstein et al. cohort and from the date of the first diagnosis in the TCGA cohort or the Zehir et al. cohort. Patients who did not progress or die were censored at the date of the last follow-up. Patients assessed as not evaluable (NE) were not included in grouping (CR/PR vs. PD or DCB vs. NDB), but were included in the subsequent survival analysis of PFS or OS.

### Tumor immunogenicity (TMB, NAL, MSI, and DDR mutations)

TMB was defined as the total number of nonsynonymous somatic mutations per megabase of the examined genome (21). For the TCGA cohort, 38 Mb was used as the estimated exome size (22). For samples sequenced using the MSK-IMPACT panel, the lengths of the exonic coverage were 0.98, 1.06, and 1.22 Mb in 341, 410, and 468 gene panels, respectively, as described in Samstein et al. (6). The cutoff value to distinguish between the high- and low-TMB groups was the median value in the NSCLC cohort and the top 20% in each cancer type in the pan-cancer cohort, as described by Samstein et al. (6). Neoantigen load (NAL) was defined as the total predicted neoantigen count as determined by Thorsson et al. (23) The MSI score of each TCGA cancer sample in this study was the MSI MANTIS (Microsatellite Analysis for Normal Tumor Instability) score obtained from a previously published study; samples with MSI MANTIS scores >0.6 were classified as MSI-H (24). Gene sets associated with DNA damage repair (DDR) pathways were obtained from Knijnenburg et al. (25). The *SMO* mutation status was annotated and the corresponding mutation samples filtered according to the cBioPortal database (**Supplementary Figure S1C**).

### Antitumor immune microenvironment and pathway enrichment analysis (GSEA and GSVA)

The RNA sequencing (RNA-seq) data [fragments per kilobase of exon model per million mapped fragments (FPKM) data] and the pan-cancer clinical data of the TCGA cohort were downloaded from the UCSC Xena data portal (https://xena.browser.net/). The immune and stromal scores represented the abundance of the immune and stromal components, respectively, which were assessed using the “estimate” package. The ESTIMATE score was the sum of the immune and stromal scores (26). We quantified the enrichment levels of 24 immune signatures in each cancer sample using single-sample gene set enrichment analysis (ssGSEA). The gene set was prepared as described by Miao et al. (27, 28). The infiltration fraction of 22 immune cells was analyzed using the CIBERSORT web portal (https://cibersort.stanford.edu/) (29). Immune-related genes and their functional classifications were sourced from Thorsson et al. (23).

Gene set enrichment analysis (GSEA) was performed between the *SMO* mutant (SMO_MUT) and *SMO* wild-type (SMO_WT) groups with the help of GSEA 4.1.0 software. Four gene sets were obtained using the Molecular Signatures Database (MSigDB) of the Broad Institute (30), including Reactome, Gene Ontology (GO) terms, and Hallmark. Gene set variation analysis (GSVA) scores were generated for all tumor samples according to the gene sets mentioned above and analyzed with the R packages “limma,” “GSEABase,” and “GSVA.” Pathways with *p*-values <0.05 were considered significantly different, and the most significant positive and negative correlations were visualized.

### Cells and reagents

The A549, Calu-1, HT-29, PC-9, SW480, and SW-620 cell lines were obtained from the American Type Culture Collection (ATCC). Calu-1 and SW480 cells were cultured in Dulbecco’s modified Eagle’s medium (DMEM; Gibco, Grand Island, NY, USA), while A549, PC-9, SW-620, and HT-29 cells were cultured in RPMI 1640 medium (Gibco). The media were supplemented with 10% fetal bovine serum (FBS; Gibco), 100 U/ml penicillin, and 100 μg/ml streptomycin (Gibco).

### Plasmid transfection

The wild-type *SMO* plasmid and three representative SMO_MUT plasmids (P687Q, A250D, and P694Lfs*84) were purchased from Fenghui Biotechnology Co. (Hunan, China). The plasmids were transfected into Calu-1 and PC-9 cells using Lipofectamine 2000 (Invitrogen, Carlsbad, CA, USA) following the manufacturer’s protocol. After 48 h, cells were harvested for the following experiments.

### Western blot, RNA extraction, cDNA synthesis, and qPCR

The cells were lysed for protein or RNA extraction and subjected to Western blotting or cDNA synthesis and quantitative polymerase chain reaction (qPCR), as previously described by us (31). The antibodies for Western blotting were as follows: mouse anti-SMO (66851-1-1g; Proteintech, Rosemont, IL, USA), rabbit anti-AKT (10176-2-AP; Proteintech), rabbit anti-p-AKT (ab38449; Abcam, Cambridge, UK), rabbit anti-IGF1R (20254-1-AP; Proteintech), rabbit anti-p-IGF1R (ab39398; Abcam), and rabbit anti-β-actin (66009-1-Ig; Proteintech). The primers used for qPCR are listed in **Supplementary Table S3**.

### Cell proliferation assay

Cells transfected with different SMO_MUT plasmids were seeded in 96-well plates at a density of 2,000 cells per well. After 0, 24, 48, and 72 h, 10 μl of the Cell Counting Kit-8 (CCK-8) solution was added to each well and incubated for 1–3 h at 37°C. The absorbance at 450 nm was measured with a microplate reader (Synergy 2; BioTek, Winooski, VT, USA). We repeated the assessment and calculated the optical density (OD) value at each time point.

### Statistical analysis

Assessment of the enrichment of specific mutated genes with a response (DCB vs. NDB) was performed using Fisher’s exact test. The association between the *SMO* status and ORR or DCB was also examined using Fisher’s exact test. The PFS and OS of SMO_MUT and SMO_WT patients were depicted using Kaplan–Meier curves and compared with a log-rank test, which were further adjusted using the Cox proportional hazards regression model for available confounding factors, including: 1) age, sex, histology, smoking history, lines of ICI treatment, TMB, PD-L1 expression, and treatment type in the NSCLC discovery cohort; 2) age, sex, smoking history, stage, lines of ICI treatment, PD-L1 expression, and treatment type in the NSCLC validation cohort; 3) age, sex, cancer type, TMB, and treatment type in the ICI cohort; 4) age, sex, ethnicity, cancer type, TMB, and stage in the TCGA cohort; and 5) sex, smoking history, cancer type, and TMB in the non-ICI cohort. The Cox proportional hazards model was then used to analyze the effect of potential prognostic factors on the PFS of patients with NSCLC in both the univariable and multivariable analyses. Differences in the tumor immunity-related markers between the SMO_MUT and SMO_WT groups were examined using the Mann–Whitney *U* test. Intergroup comparisons of the measurement data were performed using ANOVA with Bonferroni or Dunnett’s *post-hoc* test. The nominal level of significance was set at *p* < 0.05, and all statistical tests were two-sided. Statistical analyses were performed with R v.4.1.2, GraphPad Prism 5, and SPSS 19.

## Results

### SMO_MUT predicted favorable clinical outcomes for ICIs in the NSCLC cohort

**Figure 1:**
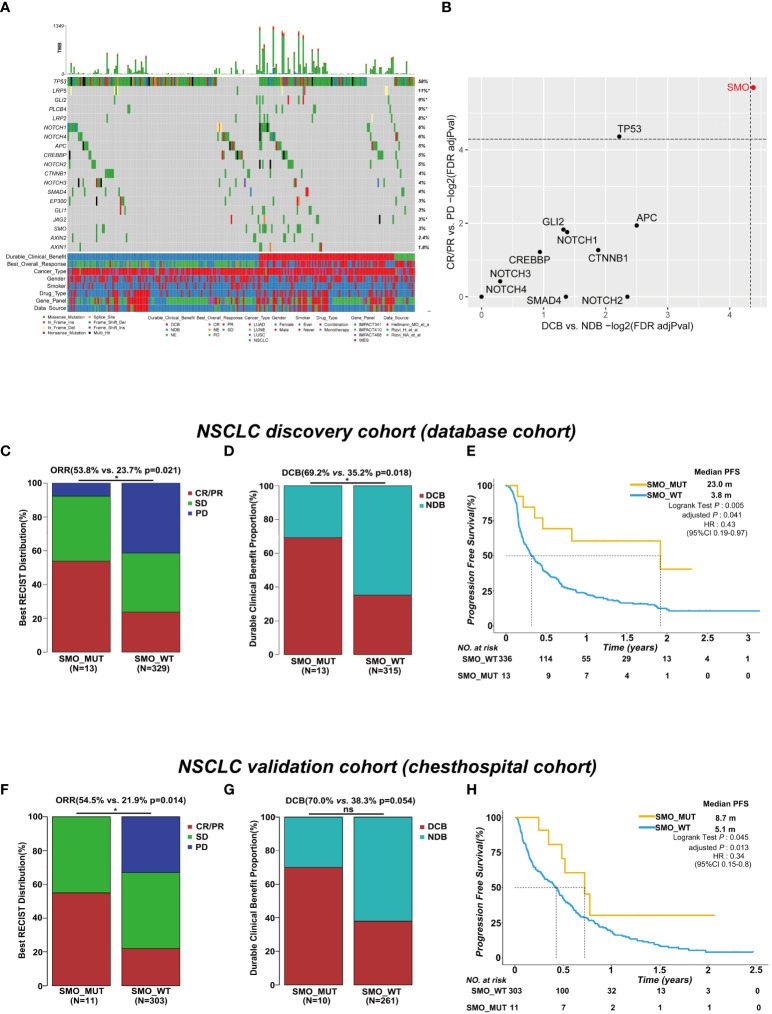
*SMO* mutation predicted favorable clinical outcomes for immune checkpoint inhibitors (ICIs) in the non-small cell lung cancer (NSCLC) cohort. **(A)** Summary of the mutational and clinical information of patients with NSCLC in the discovery cohort. Individual patients are represented in each *column*. Categories of durable_clinical_benefit (DCB), best_overall_response (BOR), cancer_type, gender, smoker, drug_type, gene_panel, and data_source are characterized. The occurrences of the selected genes in each case are represented in the OncoPrint, with the percent frequency shown. *Asterisk* denotes genes that were not covered in the MSK-IMPACT (Integrated Mutation Profiling of Actionable Cancer Targets) panel. **(B)** Associations between mutations in the developmental signaling pathway-related genes and clinical responses [complete response (CR)/partial response (PR) and DCB]. Both *dashed lines* indicate adjusted *p* = 0.05 regarding DCB and CR/PR (two-tailed Fisher’s exact test). **(C, D)** Histogram depicting the proportions of objective response rate (ORR) and DCB in *SMO* mutant (SMO_MUT) and *SMO* wild-type (SMO_WT) patients in the NSCLC discovery cohort (two-tailed Fisher’s exact test). **(E)** Kaplan–Meier survival analysis comparing the progression-free survival (PFS) between SMO_MUT and SMO_WT patients in the NSCLC discovery cohort (log-rank test). **(F, G)** Histogram depicting the proportions of ORR and DCB in SMO_MUT and SMO_WT patients in the NSCLC validation cohort (two-tailed Fisher’s exact test). **(H)** Kaplan–Meier survival analysis comparing the PFS between SMO_MUT and SMO_WT patients in the NSCLC validation cohort (log-rank test). *p < 0.05. ns, no significant.

The baseline characteristics of the patients in the discovery and validation cohorts are summarized in **Figure 1A** and **Supplementary Table S4**. We only included genes with at least five mutations in the NSCLC discovery cohort to identify statistically robust associations with response to ICI therapy. Among these genes, *SMO* mutation was the only one that was significantly enriched in both the CR/PR and DCB groups, while *TP53* mutation was only recurrent in the CR/PR group (adjusted *p* < 0.05) (**Figure 1B** and **Supplementary Table S5**), indicating that mutations in *SMO* might predict the efficacy of ICI treatment.

In the NSCLC discovery cohort (*n* = 349), there were 13 mutations in the *SMO* gene, including 7 CR/PR, 5 SD, and 1 PD, and 336 SMO_WT patients, including 78 CR/PR, 115 SD, 136 PD, and 7 NE. The ORR in SMO_MUT patients was almost twice that in SMO_WT patients [53.8% vs. 23.7%, odds ratio (OR) = 3.75, 95% confidence interval (CI) = 1.23–11.51, *p* = 0.021] (**Figure 1C**). A total of 69.2% (9/13) of patients carrying *SMO* mutations gained durable clinical benefit from ICI treatment, while only 35.2% (111/315) of patients in the wild-type group obtained benefit (OR = 4.14, 95% CI = 1.25–13.74, *p* = 0.018) (**Figure 1D**). The median PFS was 23.0 months in SMO_MUT patients, whereas it was only 3.8 months in SMO_WT patients [hazard ratio (HR) = 0.43, 95% CI = 0.19–0.97, adjusted *p* = 0.041] (**Figure 1E**). The subsequent univariate and multivariate Cox regression analyses confirmed that *SMO* mutation is an independent prognostic biomarker for patient survival (HR = 0.21, 95% CI = 0.05–0.86, *p* = 0.03) (**Supplementary Table S6**). Other independent prognostic factors included smoking history (HR = 0.62, 95% CI = 0.40–0.95, *p* = 0.029), TMB status (HR = 0.57, 95% CI = 0.39–0.82, *p* = 0.003), and PD-L1 expression (HR = 0.45, 95% CI = 0.28–0.72, *p* < 0.001).

In the NSCLC discovery cohort (*n* = 349), the ORR in TP53_MUT patients was almost twice that in TP53_WT patients (29.7% vs. 19.1%, OR = 1.79, 95% CI = 1.08–2.98, *p* = 0.025) (**Supplementary Figure S1A**). The difference between the *TP53* mutated type and the wild type was not significant either for the DCB proportion or for PFS (**Supplementary Figures S2B, C**). Hence, the role of *TP53* mutation was not explored in a further study.

In the validation cohort, we collected the data of stage IIIB/IV NSCLC patients receiving ICI treatment along with the clinical and NGS data from our center. Eleven SMO_MUT patients were included in the 314 NSCLC samples. After adjusting for age, gender, smoking history, stage, lines of ICI treatment, PD-L1 expression, and treatment type, the PFS in patients with *SMO* mutations was 8.7 months, whereas it was 5.1 months in SMO_WT patients (HR = 0.34, 95% CI = 0.15–0.8, adjusted *p* = 0.013). The ORR in SMO_MUT patients was twice the ratio found in SMO_WT patients (54.5% vs. 21.9%, *p* = 0.014), and a significant difference in the DCB proportion between the two groups was not observed, which was probably due to the limited sample size (**Figures 1F–H** and **Supplementary Table S7**).

### SMO_MUT predicted survival advantage in the pan-cancer cohort

**Figure 2:**
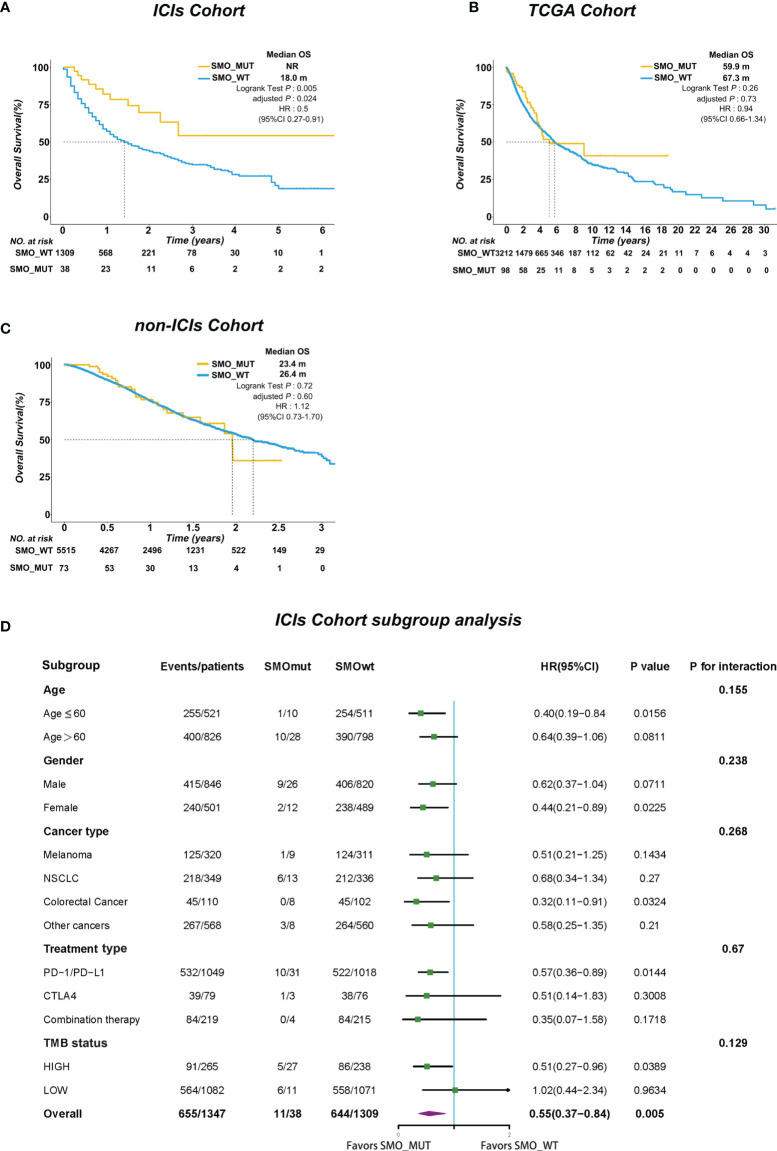
*SMO* mutation predicted survival advantage in the pan-cancer cohort. **(A–C)** Kaplan–Meier curves comparing the overall survival (OS) between *SMO* mutant (SMO_MUT) and *SMO* wild-type (SMO_WT) patients in the immune checkpoint inhibitor (ICI) cohort (Samstein et al.), The Cancer Genome Atlas (TCGA) cohort, and the non-ICI cohort (Zehir et al.; log-rank test). **(D)** Forest plot depicting the results of the subgroup analysis in the ICI cohort.

We further investigated whether the survival benefit exist in ICI-treated patients carrying *SMO* mutations in the pan-cancer cohort. In the ICI cohort (*n* = 1,347), 38 patients were carrying *SMO* mutations, including 9 melanomas, 13 NSCLCs, 1 head and neck cancer cell carcinoma, 3 bladder cancers, 8 colorectal cancers, 2 esophagogastric cancers, and 2 cancers of unknown primary origin, which comprised 2.8% of the population in the pan-cancer cohort. After adjusting for confounding factors (age, gender, cancer type, TMB, and treatment type), SMO_MUT patients achieved a significantly longer OS than SMO_WT patients [median OS not reached (NR) vs. 18 months, HR = 0.5, 95% CI = 0.27–0.91, adjusted *p* = 0.024] (**Figure 2A**). We further evaluated the survival differences between the SMO_MUT and SMO_WT groups in two non-ICI-treated pan-cancer cohorts to explore whether the OS advantage can be attributed to ICI treatment in patients carrying *SMO* mutations or was just simply due to its general prognostic impact. The OS of patients carrying *SMO* mutations appeared to be shorter without statistical significance in both the TCGA cohort (59.9 vs. 67.3 months, HR = 0.94, 95% CI=0.66–1.34, adjusted *p* = 0.73) and the non-ICI cohort (23.4 vs. 26.4 months, HR = 1.12, 95% CI = 0.73–1.70, adjusted *p* = 0.60) (**Figures 2B, C**, respectively).

In the subgroup analysis, the survival advantage of patients with *SMO* mutations receiving ICI treatment over the SMO_WT patients was prominent and consistent across each subgroup, including age, gender, cancer type, treatment type, and TMB status (all *p*_interaction_ > 0.05) (**Figure 2D**).

### Mutational landscape of *SMO* mutation distribution in the pan-cancer cohorts

**Figure 3:**
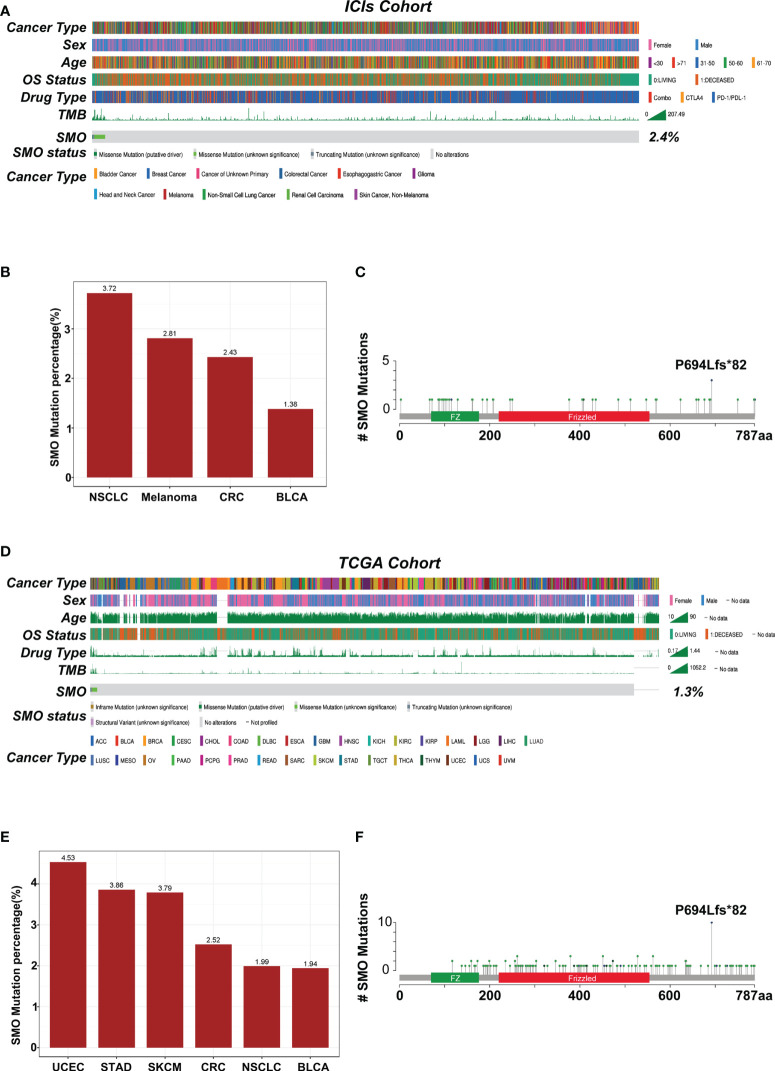
Mutational landscape of the distribution of *SMO* mutations in the pan-cancer cohorts. **(A)** Association of the *SMO* status with the clinical characteristics in the immune checkpoint inhibitor (ICI) cohort (Samstein et al.). The cancer type, sex, age, drug type, tumor mutation burden (TMB), and overall survival (OS) were annotated. Samples were sorted based on the *SMO* mutation status. **(B)** Proportion of *SMO* mutant (SMO_MUT) tumors identified in each cancer type with at least 1% proportion in the ICI cohort. *Numbers above the bar plot* indicate the alteration frequency. **(C)** Lollipop plot showing the distribution of *SMO* mutations in the ICI cohort. **(D)** Association of the *SMO* mutation status with the clinical characteristics in The Cancer Genome Atlas (TCGA) cohort. The cancer type, sex, age, microsatellite instability (MSI) score, TMB, and OS were annotated. **(E)** Proportion of SMO_MUT tumors identified in each cancer type with at least 1% proportion in the TCGA cohort. *Numbers above the bar plot* indicate the alteration frequency. **(F)** Lollipop plot showing the distribution of *SMO* mutations in the TCGA cohort.

The overall average mutation frequency of *SMO* was 2.3% (39/1,661) in the Samstein et al. cohort and was 1.2% (130/10,967) in the TCGA pan-cancer cohort, which included 32 cancer types (**Figures 3A–D** **)**. NSCLC, melanoma, and colorectal cancer were the most frequently affected in the ICI cohort, whereas uterine corpus endometrial carcinoma (UCEC), stomach adenocarcinoma (STAD), and skin cutaneous melanoma (SKCM) topped the list in the TCGA cohort (**Figures 3B–E** **)**. The cancer types with at least 1% mutation frequency are shown in **Figure 3** and were further studied in the pan-cancer TCGA cohort (six cancer types). The mutation sites of the *SMO* gene are listed in **Figures 3C–F**, which showed that P694Lfs*82 was the most frequent mutation site in both the ICI and TCGA cohorts.

The genomic distinctions between the SMO-MUT and SMO-WT groups were investigated. **Supplementary Figure S3A** shows the top 20 most frequently mutated genes and the clinical characteristics of patients in the TCGA-NSCLC cohort. The mutation rates of 8 of the top 20 most frequently mutated genes (*LRP1B*, *ZFHX4*, *NAV3*, *FLG*, *PCDH15*, *FAM135B*, *CDH10*, and *MUC17*) were significantly higher in patients with *SMO* mutations in the TCGA cohort. Among these differential genes, *TP53*, *MUC16*, *TTN*, and *RYR2* gene mutations have been correlated with antitumor immunity in the immunotherapy of various cancer types (31–33), but they do not coexist with the *SMO* mutation, indicating that *SMO* mutation might be an independent factor predicting the efficacy of ICIs (**Supplementary Figure S3B**). The coexistence between *SMO* mutation and common driver mutations was further investigated in lung adenocarcinoma (LUAD). As shown in **Supplementary Figure S3C**, no co-occurrence was found between the other driver gene mutations and the *SMO* mutation in the LUAD cohort.

### SMO expression profiles in normal human tissues and cancer tissues

**Figure 4:**
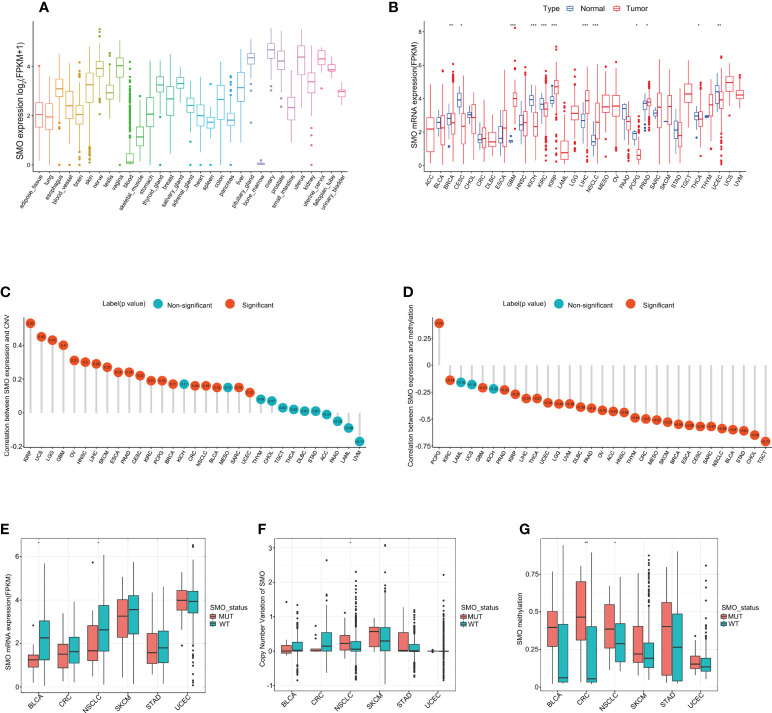
*SMO* expression profiles in normal human tissues and cancer tissues. **(A)** *SMO* expression profiles in normal human tissues in the GTEx database. **(B)** Comparison of *SMO* expression between tumor and normal samples in The Cancer Genome Atlas (TCGA) cohort. **(C, D)** Correlations between *SMO* expression and copy number variant (CNV) **(C)** and between *SMO* expression and promoter methylation **(D)** in the TCGA cohort. **(E, F)** Comparisons of the *SMO* expression **(E)** and *SMO* CNV **(F)** between the *SMO* mutant (SMO_MUT) and *SMO* wild-type (SMO_WT) tumors in different cancer types in the TCGA cohort (Mann–Whitney *U* test). **(G)** Comparison of *SMO* promoter methylation between the SMO_MUT and SMO_WT tumors in different cancer types in the TCGA cohort (Mann–Whitney *U* test). *p < 0.05, **p < 0.01, ***p < 0.001.

We analyzed the physiological gene expression levels of *SMO* across normal tissues using the GTEx dataset (**Figure 4A**). The expression levels of *SMO* were the highest in ovary tissue, but lowest in the bone marrow. Furthermore, we analyzed the expression levels of *SMO* in various cancers. All cancers expressed *SMO*, with the highest levels in kidney renal papillary cell carcinoma (KIRP) and the lowest levels in pheochromocytoma and paraganglioma (PCPG). We also compared the *SMO* expression levels between cancer samples and matched normal samples from 33 cancer types based on the TCGA data. Except for those cancers without normal tissue data available, significant differences in the expression of *SMO* were detected between tumor and normal tissue in 11 types of cancer. Among them, *SMO* was highly expressed in glioblastoma multiforme (GBM), KIRP, NSCLC, liver hepatocellular carcinoma (LIHC), and prostate adenocarcinoma (PRAD) compared with normal tissue. On the contrary, the *SMO* levels were downregulated in tumors relative to normal tissues in breast invasive carcinoma (BRCA), cervical squamous cell carcinoma and endocervical adenocarcinoma (CESC), kidney chromophobe (KICH), kidney renal clear cell carcinoma (KIRC), PCPG, thyroid carcinoma (THCA), and UCEC (**Figure 4B**). The expression of *SMO* was affected by several factors. The level of *SMO* mRNA has been negatively correlated with the methylation status of the *SMO* promoter in multiple cancer cells (34). Based on the TCGA data, we found that the copy number variant (CNV) and promoter methylation status were commonly positively and negatively correlated with *SMO* expression in multiple cancers, respectively (**Figures 4C, D** **)**. We further found that the expression of *SMO* was lower in the *SMO* mutation samples than that in the wild-type bladder urothelial carcinoma (BLCA) and NSCLC. Although the CNV was higher in NSCLC with *SMO* mutations, the higher promoter methylation in SMO_MUT tumors might have led to the lower expression of *SMO* in *SMO* mutant NSCLC (**Figures 4E–G**).

Subsequently, we investigated the mRNA expression of *SMO* in several cancer cell lines by quantitative real-time PCR (qRT-PCR). *SMO* was significantly overexpressed in A549 and was lowest in Calu-1 and PC-9 (**Supplementary Figure S4A**). P687Q and A250D were the two mutations that recurrently appeared in the NSCLC discovery cohort, while P694fs*84 was common in the MSKCC-ICI and TCGA cohorts. Thus, these three mutations were chosen as representative mutations for further study. Three different SMO_MUT plasmids were transfected into the two low-*SMO*-expression cell lines (Caku-1 and PC-9) to explore the effect of the *SMO* mutation status on their mRNA and protein expressions. All these *SMO* plasmids induced the expression of *SMO* at both the mRNA and protein levels, but the expression efficiency of the SMO_MUT plasmids was lower compared to that of the wild type, which was in line with our previous database results showing that the expression of *SMO* is lower in mutant NSCLC samples (**Supplementary Figures S4B–E**). Most of the mutant plasmids could stimulate the growth of cancer cells, but the effects were different among various mutations in different cell lines (**Supplementary Figures S4F, G**).

### Association of *SMO* mutation with enhanced tumor immunogenicity

**Figure 5:**
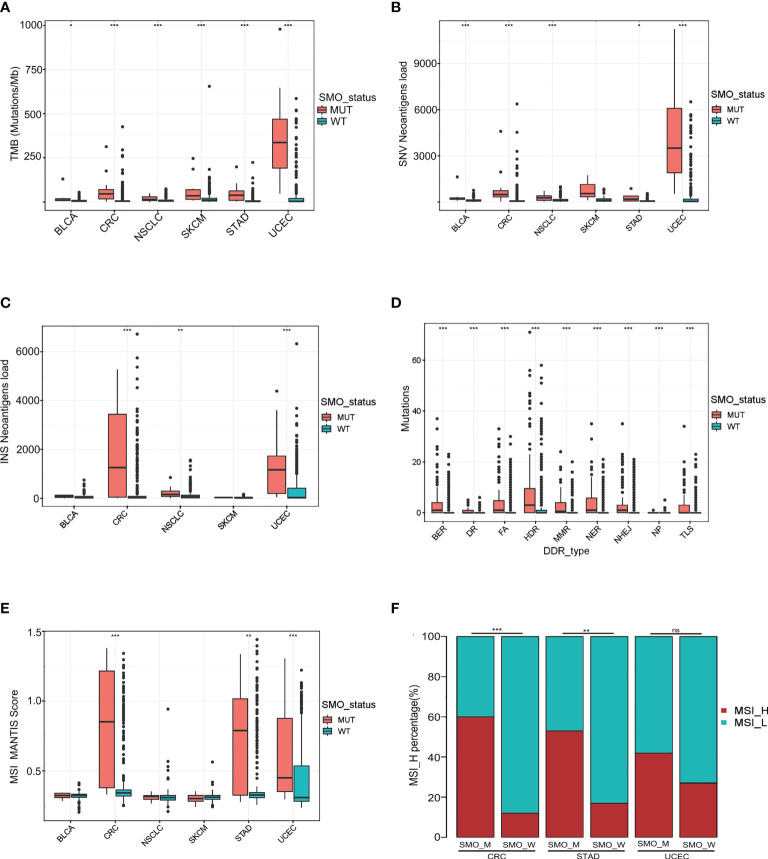
Association of *SMO* mutation with enhanced tumor immunogenicity. **(A–C)** Box plot depicting the distributions of tumor mutation burden (TMB), single nucleotide variant (SNV) neoantigen load, and insertion–deletion (INDEL) neoantigen load in *SMO* mutant (SMO_MUT) and SMO wild-type (SMO_WT) tumors in The Cancer Genome Atlas (TCGA) cohort (two-tailed Mann–Whitney *U* test). **(D)** Mutation rates of the DNA damage repair (DDR) pathway genes between the *SMO* mutant (SMO_MUT) and wild-type (SMO_WT) subgroups in the TCGA cohort (two-tailed Mann–Whitney *U* test). **(E)** Box plot depicting the distribution of the microsatellite instability (MSI) scores in SMO_MUT and SMO_WT tumors in the TCGA cohort (two-tailed Mann–Whitney *U* test). **(F)** MSI-H percentages in SMO_MUT or SMO_WT samples in colorectal cancer (CRC), stomach adenocarcinoma (STAD), and uterine corpus endometrial carcinoma (UCEC; two-tailed Fisher’s exact test). *p < 0.05, **p < 0.01, ***p < 0.001. ns, no significant.

We compared the TMB level, NAL, MSI score, and DDR gene mutations between tumors carrying *SMO* mutations and those that are *SMO* wild type in the TCGA pan-cancer cohort to investigate the underlying mechanism linking *SMO* mutation to ICI response. We found that the TMB and single nucleotide variant (SNV) NAL were synchronously higher in the majority of SMO_MUT tumors (**Figures 5A, B** **)**. The NAL of insertion–deletion (INDEL) was also upregulated in colorectal cancer (CRC), NSCLC, and UCEC (**Figure 5C**). In the cohort from Hellmann et al., an ICI cohort, the TMB and NAL were significantly higher in the SMO_MUT group (*p* < 0.05) (**Supplementary Figures S5A, B**). In the larger ICI cohort (Samstein et al.), the TMB was higher in the SMO_MUT group in multiple cancer types (**Supplementary Figure S5C**).

Defects in the DDR system lead to genome instability, which, in turn, increases the overall rate of somatic mutations. Therefore, we investigated whether the *SMO* mutation status was associated with DDR deficiency. We observed an enrichment of the DDR gene mutations in *SMO*-mutated tumors in the TCGA pan-cancer cohort, the ICI cohort, and the non-ICI cohort (**Figure 5D** and **Supplementary Figures S5D, E**).

The MSI score was even higher in the SMO_MUT samples than that in wild-type CRC, STAD, and UCEC (**Figure 5E**). The MSI-H percentage of SMO_MUT tumors was dramatically higher than that of their counterparts in CRC (60% vs. 11.6%, *p* < 0.0001), STAD (52.9% vs. 17.1%, *p* < 0.01), and UCEC (47.1% vs. 27.1%, *p* = 0.16) (**Figure 5F**).

### Relationship between *SMO* mutation and the antitumor immune microenvironment

**Figure 6:**
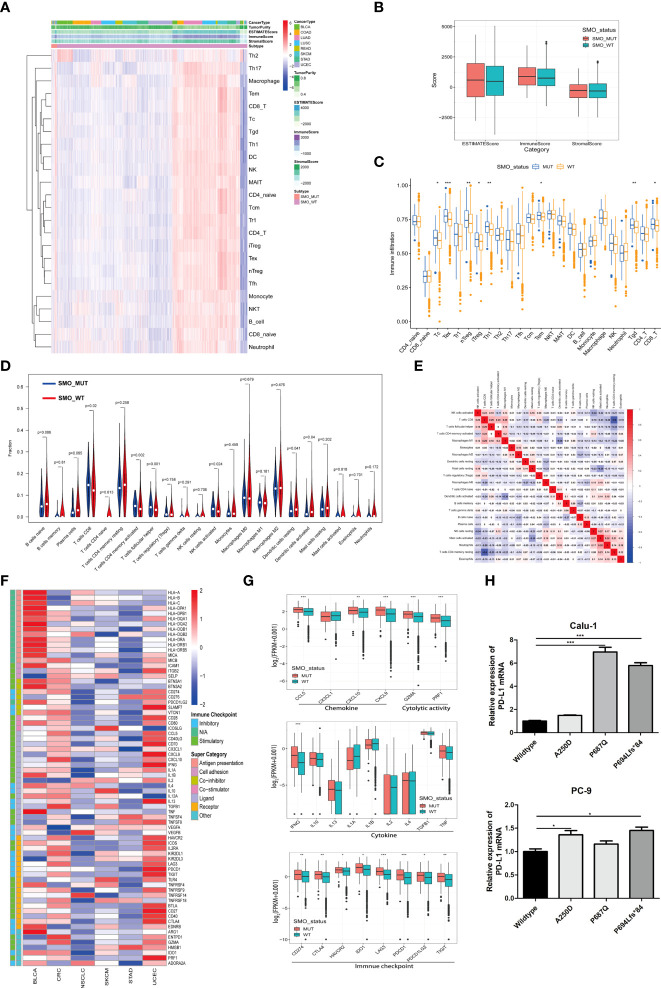
Relationship between *SMO* mutation and the antitumor immune microenvironment. **(A)** Heatmap illustrating the hierarchical clustering of immune cell signaling in pan-cancers between *SMO* mutant (SMO_MUT) and *SMO* wild-type (SMO_WT) tumors. Immune cell signaling enrichment was quantified by the single-sample gene set enrichment analysis (ssGSEA) score in each tumor sample. The stromal score, immune score, and ESTIMATE score were evaluated by ESTIMATE (Estimation of Stromal and Immune Cells in Malignant Tumor Tissues using Expression data). **(B)** Comparison of the ESTIMATE score, stromal score, and immune score in SMO_MUT and SMO_WT tumors (two-tailed Mann–Whitney *U* test). **(C)** Box plot depicting the comparison of the immune cell infiltration levels in SMO_MUT and SMO_WT tumors based on the ssGSEA scores of 24 immune cell signals (two-tailed Mann–Whitney *U* test). **(D)** Violin plot indicating the infiltration composition ratio of 22 immune cells in SMO_MUT and SMO_WT tumors. CIBERSORT was used to calculate the infiltration degree of these immune cells. **(E)** Different correlation patterns among the 22 immune cell subsets in The Cancer Genome Atlas (TCGA) pan-cancer cohort. **(F)** Heatmap depicting the log_2_-transformed fold change in the expression levels of immune-related genes across multiple cancer types (SMO_MUT *vs*. SMO_WT). *Blue* indicates downregulation and *red* indicates upregulation. **(G)** Box plot depicting the expression levels of the different types of immune-related genes, such as chemokines, cytolytic activity, immune checkpoint, and cytokine, between the SMO_MUT and SMO_WT groups (two-tailed Mann–Whitney *U* test). **(H)** Real-time PCR analysis of programmed cell death ligand 1 (PD-L1) expression after being transfected with SMO_MUT plasmids in non-small cell lung cancer (NSCLC) cell lines (one-way ANOVA). *p < 0.05, **p < 0.01, ***p < 0.001.

An increasing number of reports has indicated that the TIME plays a vital role in tumor development and recurrence (35, 36). The ESTIMATE (Estimation of Stromal and Immune Cells in Malignant Tumor Tissues using Expression data) algorithm was used to calculate the stromal and immune cell scores in six types of cancers. There was no significant difference between the SMO_MUT and SMO_WT groups in terms of the immune scores, stromal scores, and estimate scores (*p* > 0.05) (**Figures 6A, B** **)**. We then calculated the ssGSEA score of each sample, through which we could quantify the enrichment levels of 24 immune cells in every single sample to determine the enrichment of diverse immune cell compositions between the SMO_MUT and SMO_WT groups. We found that CD8 T, cytotoxic T (Tc), induced (iTreg) and natural (nTreg) regulatory T cells (Tregs), exhausted T (Tex), T helper 1 (Th1), effector memory T (Tem), and gamma delta T (Tgd) cells were more abundant in the SMO_MUT group (**Figure 6C**). We further systematically depicted the detailed immunocyte compositions of all patients in the TCGA cohort by extracting and processing the signature GEPs using the CIBERSORT method. As described in the ssGSEA results, the fractions of CD8^+^ T cells, activated memory CD4^+^ T cells, follicular helper T (Tfh) cells, and activated natural killer (NK) cells were dramatically increased in the mutation samples (*p* < 0.05). The results from the correlation matrix revealed that CD8^+^ T cells had the strongest positive correlation with activated memory CD4^+^ T cells, Tfh cells, activated NK cells, and M1 macrophages, all of which might work together to enhance antitumor immunity (**Figures 6D, E** **)**.

A general upregulation of the stimulatory immunomodulators was observed in SMO_MUT tumors (**Figure 6F**). The expression levels of the cytolytic activity-related genes (*GZMA* and *PRF1*), the chemokine-related genes (*CCL5*, *CXCL9*, and *CCL10*), the checkpoint-related genes (*CD274*, *CTLA-4*, *HAVCR2*, *IDO1*, *LAG3*, *PDCD1*, *PDCD1LG2*, and *TIGIT*), and *IFNG* were upregulated in SMO_MUT tumors (all *p* < 0.05) (**Figure 6G**). The overexpression of three SMO_MUT plasmids in the Calu-1 and PC-3 cell lines led to the upregulation of the expression of PD-L1 to some degree (**Figure 6H**).

The profiles indicated that the *SMO* mutation status, to some extent, affected the immune cell infiltration process and immune-related gene expression, which played a vital role in the immune–oncological interactions.

### Correlation between the *SMO* mutation status and signaling pathways

**Figure 7:**
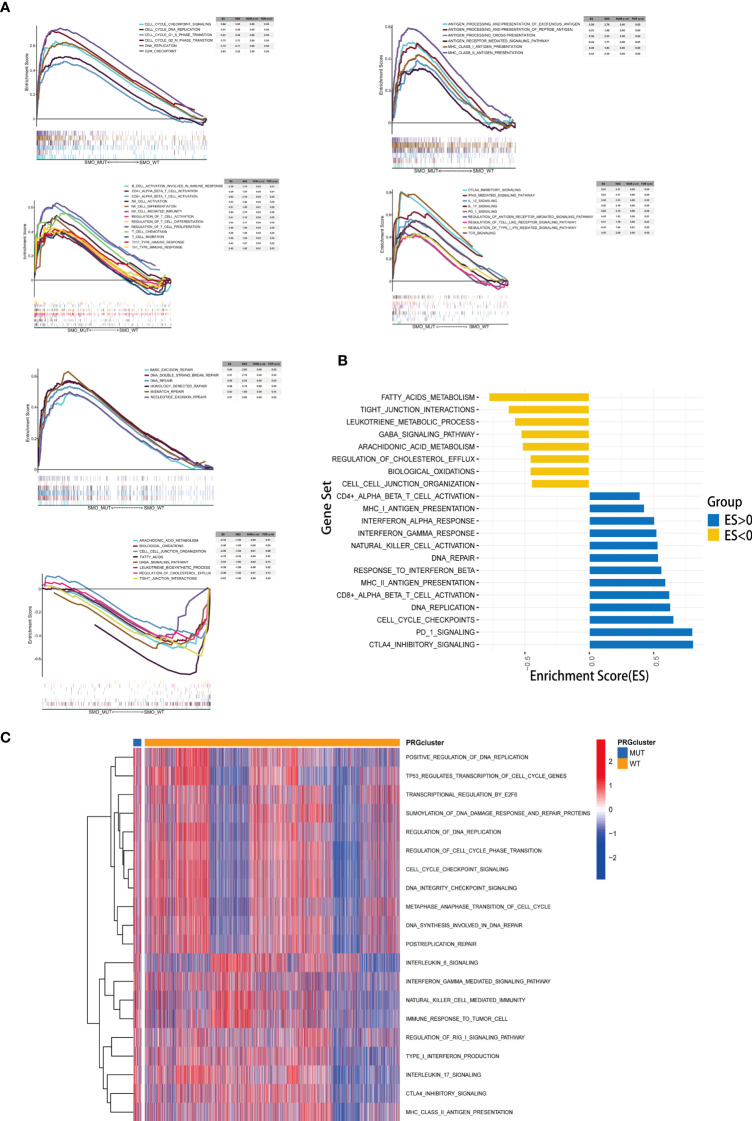
Correlation between the *SMO* mutation status and signaling pathways. **(A)** Gene set enrichment analysis (GSEA) of Gene Ontology biological process (GO-BP), Hallmark, and Reactome gene sets downloaded from MSigDB. In each panel, the gene sets marked on the *left* were enriched in the *SMO* mutant (SMO_MUT) group, while the pathways marked on the *right* were enriched in the *SMO* wild-type (SMO_WT) group. Each run was performed with 1,000 permutations. **(B, C)** Differences in pathway activities scored using GSEA **(B)** and gene set variation analysis (GSVA) **(C)** between the SMO_MUT and SMO_WT tumors in The Cancer Genome Atlas (TCGA) cohort. Enrichment results with significant associations between the SMO_MUT and SMO_WT tumors are shown. In **(B)**, the *blue bar* indicates that the enrichment score (ES) of the pathway is more than 0, while the *yellow bar* indicates that the ES of the pathway is less than 0.

The results of the enrichment analysis showed that several pathways significantly varied between SMO_MUT and SMO_WT tumors, including metabolism, intercellular interaction, immune function, and other biological functions. Cholesterol efflux, fatty acid metabolism, biological oxidation, cell–cell communication, cell–cell junction organization, and tight junction interactions were downregulated in SMO_MUT tumors (all *p* < 0.05). Gene sets such as cell cycle and DNA replication, DNA damage repair, antigen processing and presentation, NK/CD4^+^/CD8^+^ T-cell activation, and interferon gamma response were upregulated in SMO_MUT tumors (all *p* < 0.05) (**Figures 7A, B** and **Supplementary Table S8**). We also performed GSVA to calculate the ssGSEA score of each tumor sample in the above-mentioned gene sets (including cell cycle and DNA replication, DNA damage repair, and immunology gene sets). Similar to the results of the GSEA, cell cycle and DNA replication; DNA damage repair response; several immune cell-related signaling pathways, including NK cells; and immune factor-related pathways, such as interferon-γ and interleukin-6, were upregulated in SMO_MUT tumors (**Figure 7C** and **Supplementary Table S9**).

**Figure 8:**
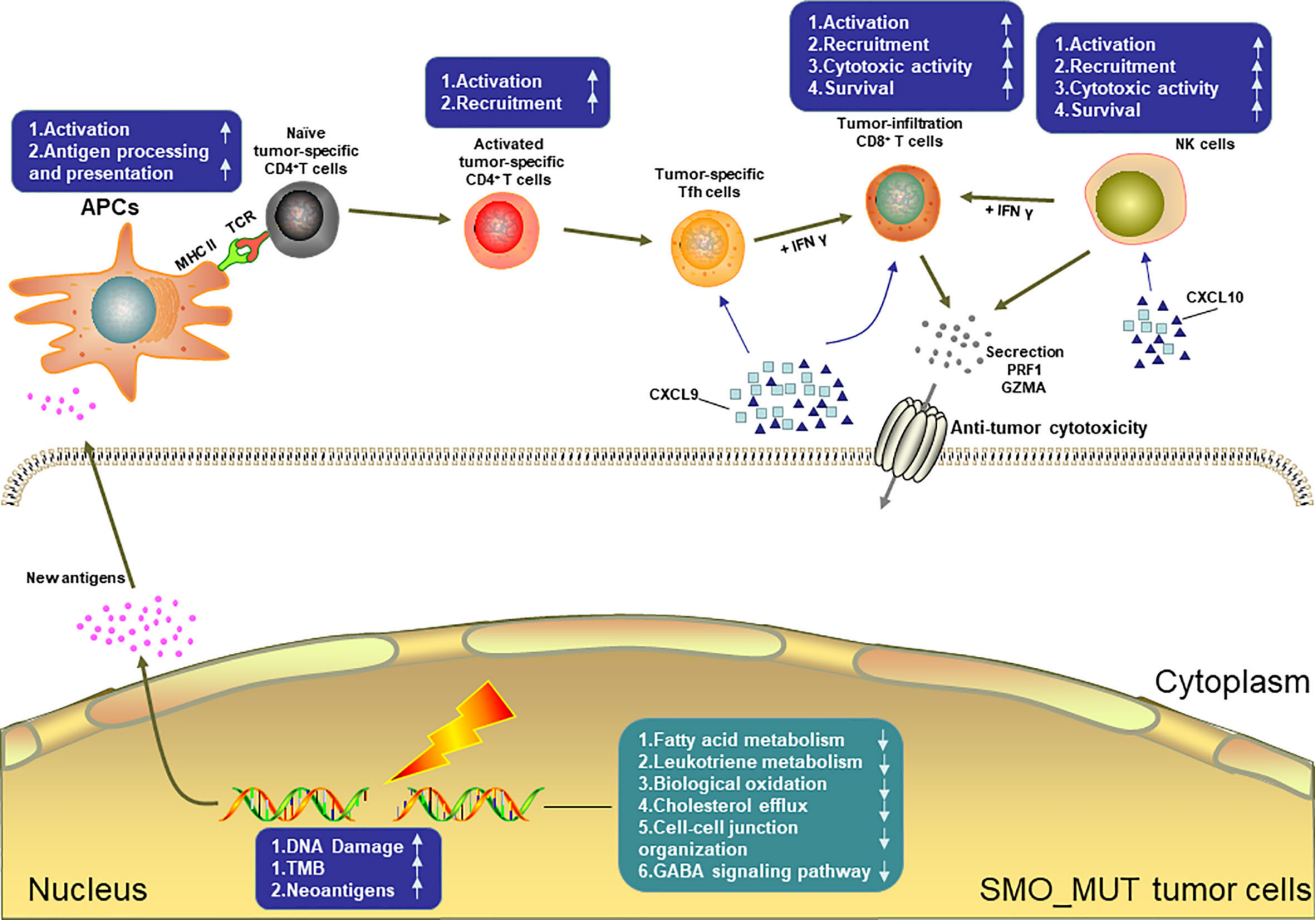
Possible tumor immune microenvironment in *SMO* mutant tumors. *APCs*, antigen-presenting cells; *NK* cell, natural killer cell.

**Figure 8** summarizes the possible TIME of SMO_MUT tumors according to the results of the pathway enrichment analysis.

## Discussion

The Hedgehog signaling pathway plays an important role in embryonic development and cell proliferation and differentiation, but the reactivation of this pathway in adult tissues has been linked to various solid neoplasms (37). The dysregulated Hedgehog pathway has an important role in tumorigenesis in certain cancer types, such as basal cell carcinoma (BCC), medulloblastoma (MB), and ovarian, breast, prostate, and lung cancers (38, 39). Notably, there is increasing evidence showing that oncogenic Hedgehog signaling regulates immunosuppressive mechanisms, including the activation of immunosuppressive cytokines, upregulation of immune checkpoints, or expansion and chemotactic recruitment of immunosuppressive cells such as Tregs and MDSCs (40–43). *SMO*, a G-protein-coupled receptor-like molecule, positively regulates Hedgehog signaling. The latest study has indicated that *SMO* is involved in the regulation of the oncogenic IGF1R/AKT signaling axis and is unrelated to canonical Hedgehog signaling in diffuse large B-cell lymphoma (DLBCL) (44). The activity of the IGF-1/IGF1R signaling pathway affects the efficiency of PD-1 inhibitors (45). However, in our work, the results of the enrichment analysis did not show a close relationship between the IGF1R pathway and *SMO* mutation. We transiently transfected Calu-1 and PC-3 cells with SMO_MUT plasmids, but we did not observe the activation of the IGF1R/AKT pathway. This result indicated that *SMO* mutations might not directly affect the IGF1R/AKT pathway in lung cancer cells (**Supplementary Figures S6A–D**). Thus, it was meaningful to discuss the role of *SMO* in antitumor immunity independent of its canonical Hedgehog signaling role. However, the association between the genomic alterations of *SMO* and response to ICIs is still unclear, which requires elucidation. This is the first study to perform a comprehensive analysis of the relationship between the *SMO* gene mutation status and the clinical outcomes of patients who received ICI-treatment across multiple cancer types.

SMO_MUT patients achieved significantly longer OS than did SMO_WT patients under ICI treatment (**Figure 2A**). The subgroup analysis showed that the survival advantage of SMO_MUT over SMO_WT was prominent and consistent across the subgroups of age, gender, treatment type, TMB status, and cancer type (**Figure 2D**). However, survival analysis revealed that the median OS tended to be longer in the SMO_WT group in both the TCGA and non-ICI cohorts than in patients with *SMO* mutations, but without statistical significance (**Figures 2B, C** **)**. Our *in vitro* results indicated that *SMO* mutations could induce the expression of PD-L1 (**Figure 6H**) and promote the growth of cancer cells (**Supplementary Figures S4F, G**). These findings indicate that *SMO* mutation might have a worse prognostic impact on cancer patients, but ICI treatment in patients with *SMO* mutations might overturn the harmful prognostic impact into a longer OS and better clinical outcomes.

The immunogenicity of the tumor is the basis for the initiation of the antitumor immune response. The accumulation of nonsynonymous mutations results in the production of more neoantigen or special unique tumor antigens and enhances the immunogenicity to increase the immune killing ability of T cells regarding tumor cells (46). Neoantigens come from different mutation types and can be classified into SNV and INDEL neoantigens. In this study, we noticed that SMO_MUT tumors were associated with a higher mutation burden and NAL of SNV. MSI-H is another pan-cancer biomarker for ICIs approved by the FDA (47). The frequency of MSI-H is about 4% in pan-cancers (48), and it is clustered in UCEC, CRC, and STAD while being rarely detected in other cancers (49). It is worth noting that the mutation frequency of *SMO* was the highest in these cancers, and the MSI score and the MSI-H proportion were higher in the SMO_MUT group in these three cancers compared to the wild type (**Figures 5E, F** **)**. Previous studies have shown that mutations in the DDR pathway might serve as a potential predictive biomarker for ICI treatment and improve the clinical outcomes of ICI treatment (50). Here, we found that SMO_MUT tumors were always accompanied by DDR pathway mutations compared to their counterparts (**Figure 5D** and **Supplementary Figures S5D, E**). Considering the mutation frequency and the correlation of *SMO* mutation with TMB, NAL, and MSI, the survival benefit of *SMO* mutation independent of TMB, PD-L1, and smoking history (**Figure 1E** and **Supplementary Table S5**) suggests that the *SMO* mutation status could be a complement biomarker to TMB for predicting response to ICIs.

Another key finding of this study was that the *SMO* mutation status was highly associated with immune infiltration. Interestingly, although the total infiltration enrichment (ESTIMATE score) was similar between the SMO_MUT and SMO_WT tumors, the cell fraction of immune cells was distinct. CD8^+^ T cells, activated memory CD4^+^ T cells, Tfh cells, and activated NK cells were more likely to be enriched in SMO_MUT tumors (**Figures 6D, E** **)**. In recent years, studies have shown that GEPs can be used as novel potential predictors for the efficacy of immunotherapy (7, 51). Among them, the expression levels of *CD8A*, *CD8B*, *GZMA*, *GZMB*, and *PRF1* were used to evaluate the infiltration levels of tumor cytotoxic T lymphocytes (CTLs), and a high-CTL-infiltration group has been related to significantly prolonged survival time of patients receiving immunotherapy (7). Additionally, chemokines such as CXCL9 and CXCL10 can help recruit CD8^+^ T cells to enhance immune infiltration and antitumor immunity (52). Therefore, the high expression of chemokines (such as CXCL10 and CXCL9) and molecules related to the cytolytic activity (*GZMA* and *PRF1*) might be one of the reasons for the efficacy of ICIs being better in patients with *SMO* mutation than in SMO_WT patients (**Figure 6G**).

Interferon gamma (INF-γ) can reduce the infiltration of Tregs, thereby enhancing the antitumor immune effect (53). Lipid metabolism disorders can influence the TIME; for example, cholesterol and its biosynthetic intermediates have profound effects on multiple aspects of immunity. These include a role in macrophage phagocytosis (54), inflammasome activation (55), and the antitumor responses of CD8^+^ T cells (56). After GSEA and GSVA, we found that multiple immune and biological metabolism-related processes were associated with the mutation status of *SMO*. Primarily, we found that the cell cycle- and DNA replication-related gene sets were overexpressed in SMO_MUT tumors compared to their counterparts, which might partially explain the higher TMB and NAL in the former. Furthermore, we found that multiple segments of antitumor immunity were activated in SMO_MUT patients, including antigen processing and presentation, NK/CD4^+^/CD8^+^ T-cell activation, and INF-γ response. In contrast, cholesterol efflux, fatty acid metabolism, biological oxidation, cell–cell communication, cell–cell junction organization, and tight junction interactions were significantly downregulated in patients with *SMO* mutations (**Figures 7A, B** **)**.

This retrospective analysis had several limitations. Firstly, although we applied the Cox proportional hazards model to correct the survival curve in order to reduce the bias caused by the covariates, the influencing factors available in each cohort were different; moreover, the cancer types and sample sizes in this study were limited. Therefore, the clinical significance of *SMO* mutation needs to be verified in larger future prospective trials. Additionally, the possible TIME and molecular mechanisms of *SMO* mutation were demonstrated based on GSEA, which requires further validation in molecular *in vitro* or *in vivo* research. Finally, the mutation frequency was around 1%–2%; hence, the mutation samples were limited for classification into truncated or non-truncated mutations according to the mutation effect to evaluate the prognostic discrepancy between different mutation types.

Importantly, these limitations did not preclude the favorable clinical outcomes derived from immunotherapy in SMO_MUT patients. Unlike the continuous variables, e.g., TMB or PD-L1 expression, mutations in *SMO* can be easily detected using NGS and can clearly classify patients into two groups associated with response to immunotherapy. To the best of our knowledge, this is the first study performing a comprehensive analysis of the value of *SMO* mutation in a wide range of cancers. This research provided valuable new insights into the role of *SMO* mutation in cancer immunotherapy and revealed the association between *SMO* mutation and important immunological indicators (immune cell infiltration, immune modulators, and immune biomarkers), which might be beneficial for the understanding of the potential mechanisms linking the *SMO* mutation status and the immune system.

## Conclusion

Our study first demonstrated that the *SMO* mutation status is an independent prognostic factor for predicting the efficacy of ICIs in patients with cancer. With the help of public data and bioinformatics methods, we performed a rather comprehensive analysis of the *SMO* mutation status and the TIME. Prospective clinical trials and exploration of the related molecular mechanisms are warranted to further study the predictive role of *SMO* mutation.

## Data availability statement

The original contributions presented in the study are included in the article/**Supplementary Material**. Further inquiries can be directed to the corresponding author.

## Ethics statement

The studies involving human participants were reviewed and approved by Institutional Ethical Committee for Clinical Investigation of Shanghai Chest Hospital (no. IS2003). Written informed consent for participation was not required for this study in accordance with the national legislation and the institutional requirements.

## Author contributions

WJ, XN, and SL: Study design. WJ, YY, ZL, and XN: Data analysis and interpretation. WJ and XN: Writing of the manuscript. YY, ZL, LG, and SL: Revision of the manuscript. WJ, YY, and ZL: Statistical analysis. All authors contributed to the article and approved the submitted version.

## Funding

This work was supported by the National Natural Science Foundation of China (82030045 to SL, 82002423 to WJ, and 81972187 to XN); Technology Innovation Program of Shanghai (19411950500 to SL); Projects of the Committee of Shanghai Science and Technology (19YF1407300 to WJ and 19ZR1449800 and 20Y11913700 to XN); Project of Shanghai Talent Development Fund (2019074 to XN); Guangdong Association of Clinical Trials (GACT)/Chinese Thoracic Oncology Group (CTONG) and Guangdong Provincial Key Lab of Translational Medicine in Lung Cancer (2017B030314120 to XN); and Beijing CSCO (Sisco) Clinical Oncology Research Grant (Y-HS202101-0205 to XN).

## Acknowledgments

We would like to thank the TCGA research network, cBioPortal, and MsigDB for providing the data analyzed in this study. We also thank all the authors for making their valuable research data public.

## Conflict of interest

The authors declare that the research was conducted in the absence of any commercial or financial relationships that could be construed as a potential conflict of interest.

## Publisher’s note

All claims expressed in this article are solely those of the authors and do not necessarily represent those of their affiliated organizations, or those of the publisher, the editors and the reviewers. Any product that may be evaluated in this article, or claim that may be made by its manufacturer, is not guaranteed or endorsed by the publisher.

## Supplementary material

Supplementary Figure 1 Flowchart of the study design. **(A)** The NSCLC cohort included 3 published studies to filter the target mutations and evaluate the clinical effect, and the primary end point was PFS, ORR, and DCB. (NSCLC discovery cohort included Rizvi NA et al, Hellmann MD et al. and Rizvi H et al, NSCLC discovery cohort was Shanghai Chest hospital cohort). **(B)** The pan-cancers ICIs/non-ICIs cohort to consolidate the prognostic value of *SMO* mutation in pan-cancers, and the primary end point was OS. (Samstein RM et al/Zehir A et al). **(C)** With help of TCGA pan-cancer dataset to evaluate the relationship between *SMO* mutation status and TIME.

Supplementary Figure 2 Association between *TP53* mutation and clinical outcomes in the NSCLC cohort. **(A, B)** Histogram depicting proportions of ORR and DCB in TP53_MUT and TP53_WT patients in the NSCLC discovery cohort (two-tailed Fisher’s exact test). **(C)** The Kaplan-Meier survival analysis comparing PFS between TP53_MUT and TP53_WT patients in the NSCLC discovery cohort (log-rank test). (**p* < 0.05, ***p <*0.01, *** *p <*0.001).

Supplementary Figure 3 Landscape of somatic mutations and characteristics of *SMO* mutations in the TCGA cohort. **(A)** Top 20 most frequently mutated genes in the TCGA cohort. The alteration type and *SMO* status are annotated. **(B)** The co-current and mutually exclusive relationship between the top 20 mutations and *SMO* mutations in TCGA-NSCLC. **(C)** The co-current and mutually exclusive relationship between the common driver mutations and *SMO* mutations in TCGA-LUAD. The significant differences are labeled with dot. (Significance was calculated using Fisher’s exact test). (*p* < 0.05, **p <*0.001).

Supplementary Figure 4 Associations between SMO mutation status and SMO expression and cell proliferation. **(A)** mRNA levels of *SMO* in different cells determined by real-time PCR (qRT-PCR) quantitative. **(B, C)** Effect of different SMO mutations on SMO expression at the mRNA level in Calu-1 and PC-9 cells (one way ANOVA test). (D, E) Effect of different *SMO* mutations on *SMO* expression at the protein level in Calu-1 and PC-9 cells. **(F, G)** *SMO* mutations stimulate the growth of Calu-1 and PC-9 cells (two-way ANOVA test). (**p* < 0.05, ***p <*0.01, ****p <*0.001).

Supplementary Figure 5 Associations between *SMO* mutation status and TMB, Neoantigens load and DDR pathway gene mutation counts in the ICIs cohort and non-ICIs cohort. **(A, B)** Boxplot depicting the distribution of TMB, and neoantigen between SMO_MUT and SMO_WT in the Hellmann MD et al. cohort. **(C)** Boxplot depicting the distribution of TMB between SMO_MUT and SMO_WT in the ICIs cohort (Samstein et al). **(D, E)** Mutation amounts of DDR pathway gene mutation counts between *SMO*-mutated and wild-type subgroups in the ICIs cohort (Samstein et al) and the non-ICIs cohort (Zehir A et al). (**p* < 0.05, ***p <*0.01, ****p <*0.001, two-tailed Mann-Whitney U test).

Supplementary Figure 6 Association between *SMO* mutation and IGR1R/AKT pathway. **(A, B)** GSEA analysis of IGF1R related signaling activity between the *SMO*-mutated and the wild-type tumors. **(C, D)** Western blot analysis of different *SMO* mutations on the IGF1R/AKT pathway activity.

Supplementary Table 8 Significant pathways detected by gene set enrichment analysis. (SMO_MUT vs SMO_WT tumors). (*p*<0.05 were listed).

Supplementary Table 9 Differential GSVA score between SMO_MUT and SMO_WT tumors in TCGA pan-cancers. (*p*<0.05 were listed).

## Glossary

**Table:**

*TCGA Cancer types*	
BLCA	bladder urothelial carcinoma
CRC	colorectal carcinoma
NSCLC	non-small cell lung cancer
SKCM	skin cutaneous melanoma
STAD	stomach adenocarcinoma
UCEC	uterine corpus endometrial carcinoma
*Immune cell subtype analyzed in ssGSEA*	
B_cell	B lymphocyte
CD4_naive	naive CD4^+^ T cells
CD8_naive	naive CD8^+^ T cells
DC	dendritic cells
iTreg	induced regulatory T cells
MAIT	mucosal-associated invariant T cells
NK	natural killer cells
NKT	natural killer T cells
nTreg	natural regulatory T cells
Tc	cytotoxic T cells
Tcm	central memory T cells
Tem	effector memory T cells
Tex	exhausted T cells
Tgd	γδT cells
Tfh	follicular helper T cells
Th1	T helper 1 cells
Th2	T helper 2 cells
Th17	T helper 17 cells
Tr1	type 1 regulatory T cells
*Others*	
CI	confidence interval
CR	complete response
CTLA-4	cytotoxic T lymphocyte antigen-4
DCB	durable clinical benefit
FPKM	fragments per kilobase of exon model per million mapped fragments
HR	hazard ratio
ICIs	immune checkpoint inhibitors
MSI-H	high microsatellite instability
NAL	neoantigen load
OR	odds ratio
ORR	objective response rate
OS	overall survival
PD	progressive disease
PD-L1	programmed cell death ligand 1
PFS	progression-free survival
NE	not evaluable
PR	partial response
RECIST	Response Evaluation Criteria in Solid Tumors
SD	stable disease
TCGA	The Cancer Genome Atlas
TIME	tumor immune microenvironment
SMO_MUT	SMO mutant
SMO_WT	SMO wild type
TMB	tumor mutation burden
WES	whole-exome sequencing
