# Supplementary figures and images for: SMO mutation predicts the effect of immune checkpoint inhibitor: From NSCLC to multiple cancers
Source: Front Immunol. 2022 Nov 3;13:955800. doi: 10.3389/fimmu.2022.955800 (PMC9669061; doi:10.3389/fimmu.2022.955800)

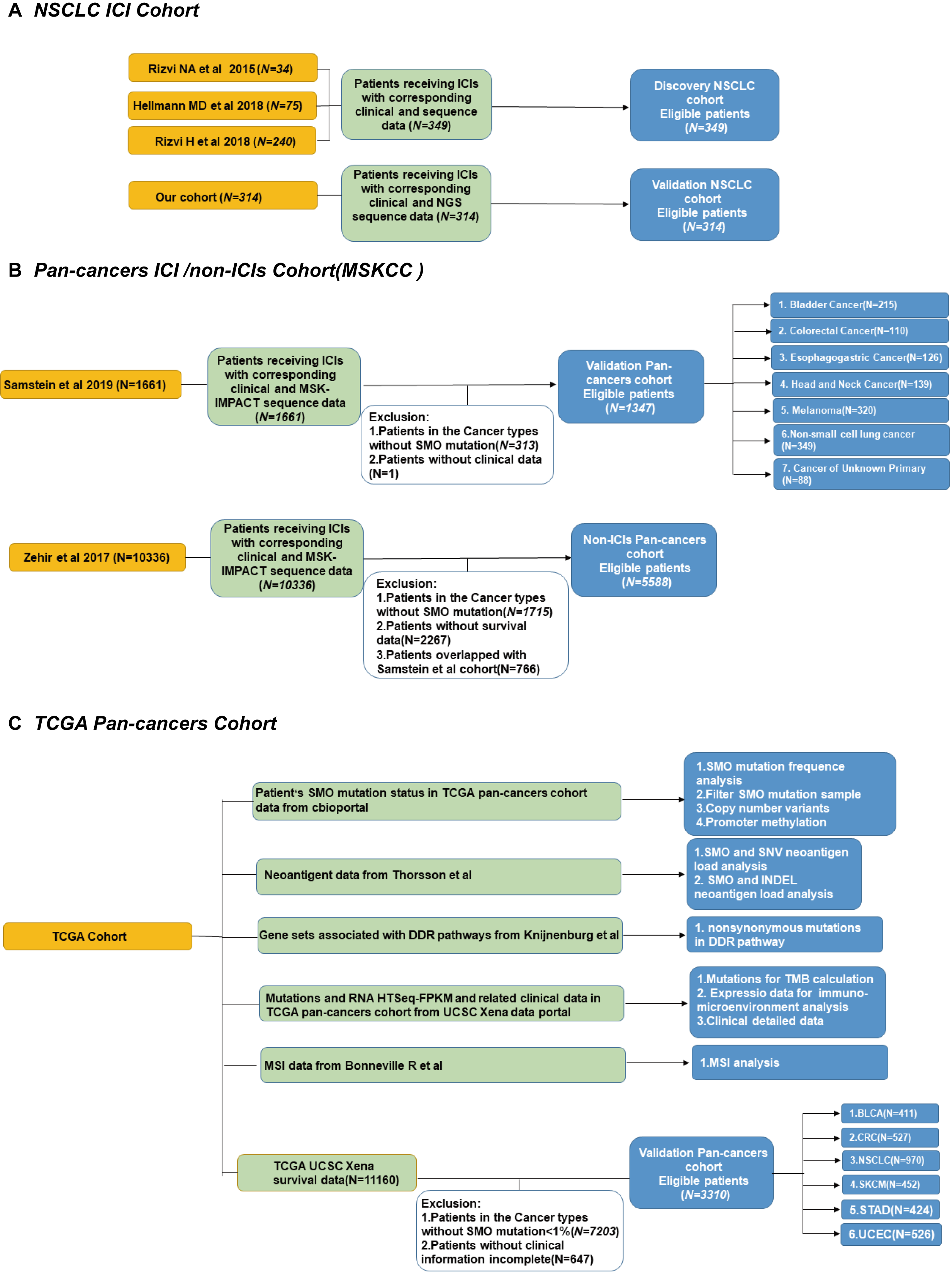

Supplement: Supplementary Figure 1 — Flowchart of the study design. (A) The NSCLC cohort included 3 published studies to filter the target mutations and evaluate the clinical effect, and the primary end point was PFS, ORR, and DCB. (NSCLC discovery cohort included Rizvi NA et al, Hellmann MD et al. and Rizvi H et al, NSCLC discovery cohort was Shanghai Chest hospital cohort). (B) The pan-cancers ICIs/non-ICIs cohort to consolidate the prognostic value of SMO mutation in pan-cancers, and the primary end point was OS. (Samstein RM et al/Zehir A et al). (C) With help of TCGA pan-cancer dataset to evaluate the relationship between SMO mutation status and TIME. [file Image_1.tif]

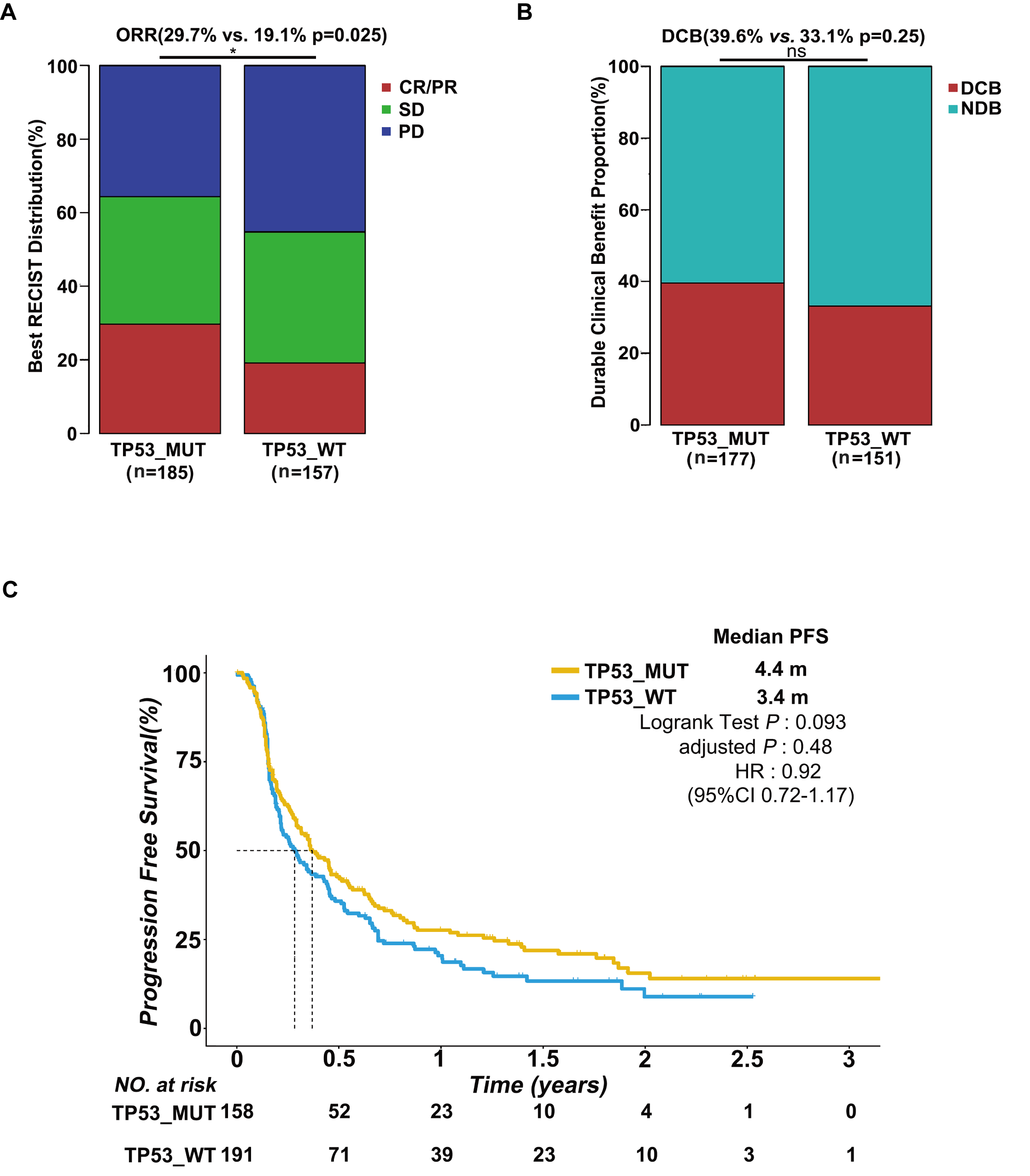

Supplement: Supplementary Figure 2 — Association between TP53 mutation and clinical outcomes in the NSCLC cohort. (A, B) Histogram depicting proportions of ORR and DCB in TP53_MUT and TP53_WT patients in the NSCLC discovery cohort (two-tailed Fisher’s exact test). (C) The Kaplan-Meier survival analysis comparing PFS between TP53_MUT and TP53_WT patients in the NSCLC discovery cohort (log-rank test). (*p < 0.05, **p <0.01, *** p <0.001). [file Image_2.tif]

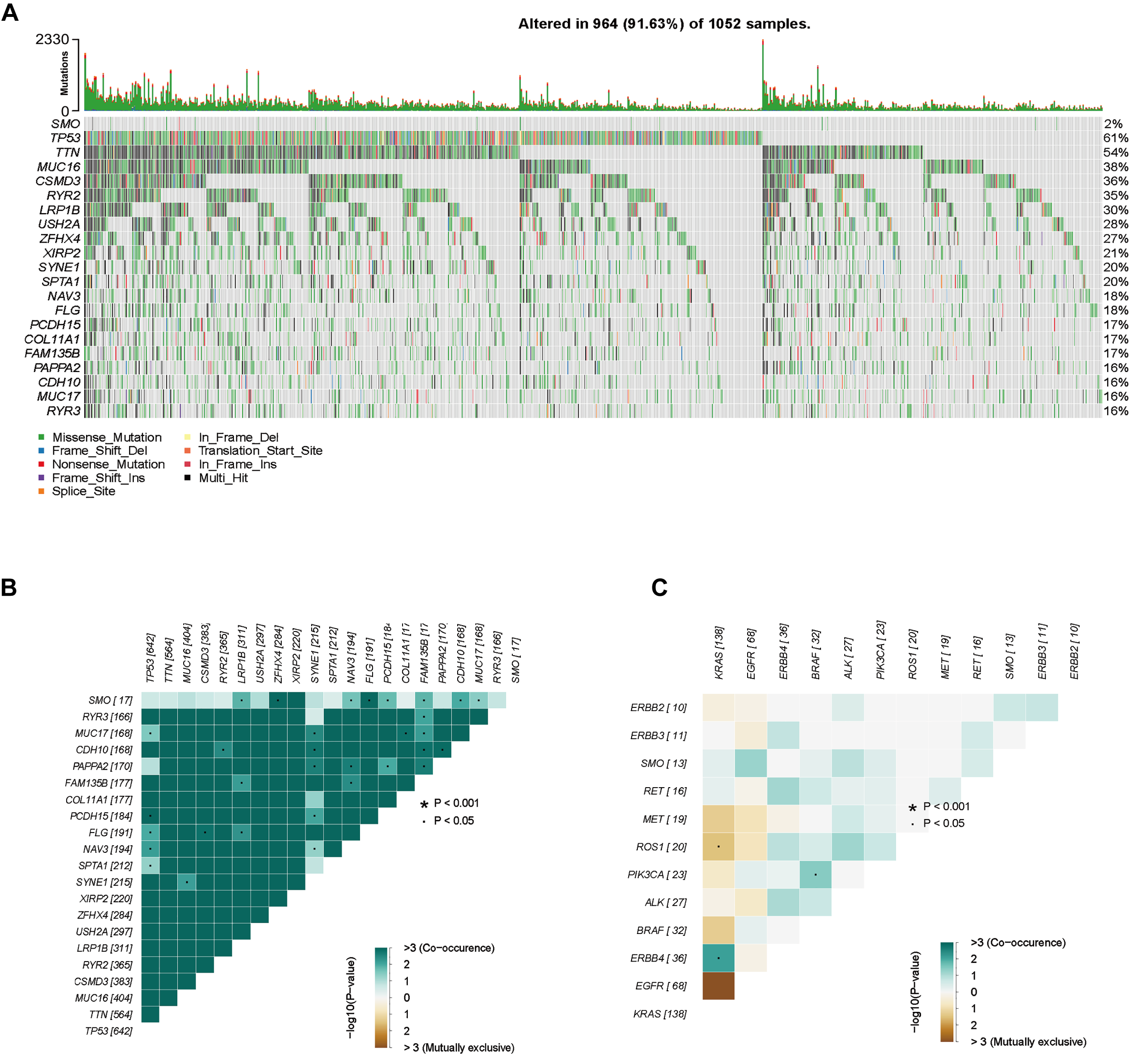

Supplement: Supplementary Figure 3 — Landscape of somatic mutations and characteristics of SMO mutations in the TCGA cohort. (A) Top 20 most frequently mutated genes in the TCGA cohort. The alteration type and SMO status are annotated. (B) The co-current and mutually exclusive relationship between the top 20 mutations and SMO mutations in TCGA-NSCLC. (C) The co-current and mutually exclusive relationship between the common driver mutations and SMO mutations in TCGA-LUAD. The significant differences are labeled with dot. (Significance was calculated using Fisher’s exact test). (p < 0.05, *p <0.001). [file Image_3.tif]

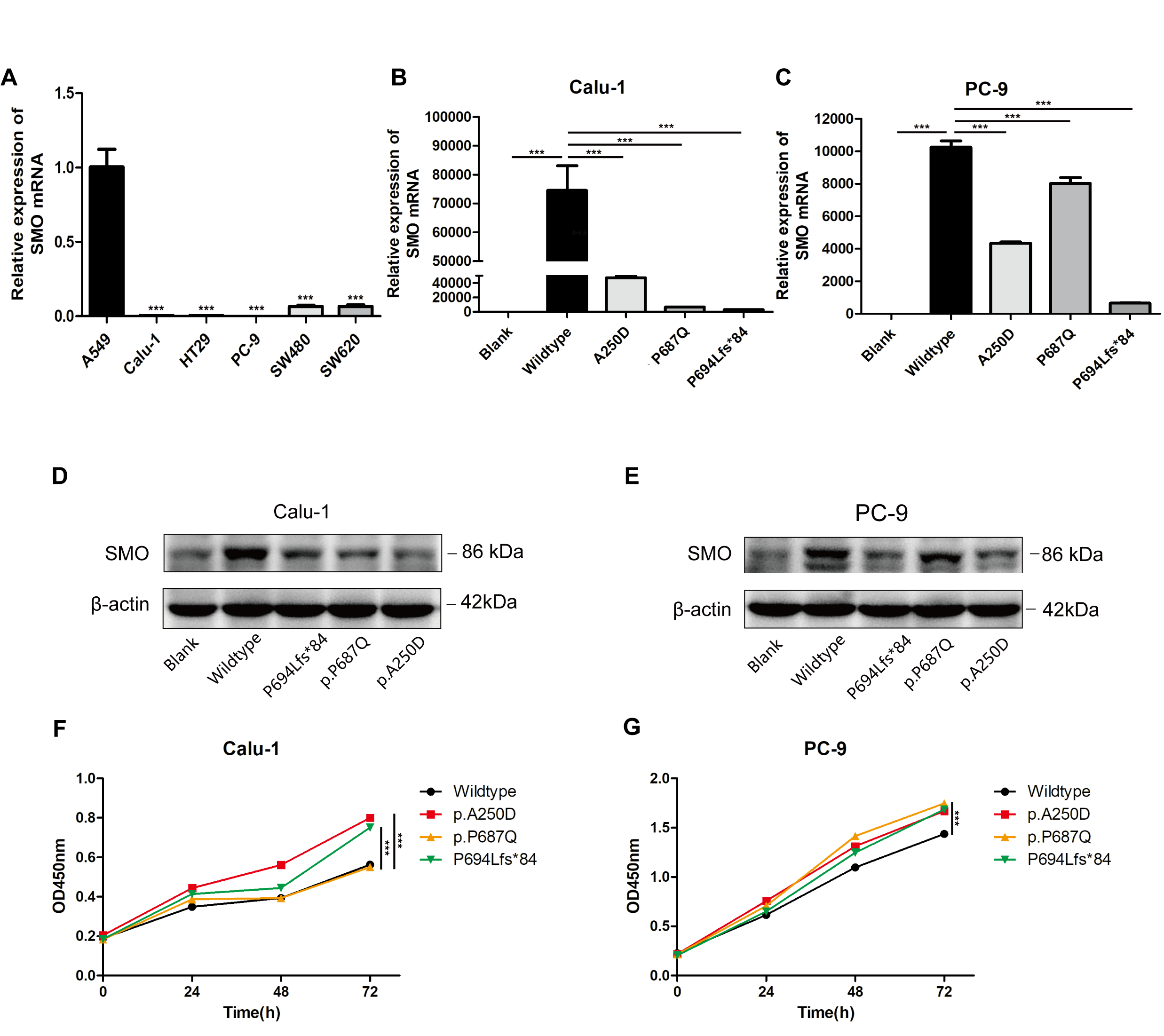

Supplement: Supplementary Figure 4 — Associations between SMO mutation status and SMO expression and cell proliferation. (A) mRNA levels of SMO in different cells determined by real-time PCR (qRT-PCR) quantitative. (B, C) Effect of different SMO mutations on SMO expression at the mRNA level in Calu-1 and PC-9 cells (one way ANOVA test). (D, E) Effect of different SMO mutations on SMO expression at the protein level in Calu-1 and PC-9 cells. (F, G) SMO mutations stimulate the growth of Calu-1 and PC-9 cells (two-way ANOVA test). (*p < 0.05, **p <0.01, ***p <0.001). [file Image_4.tif]

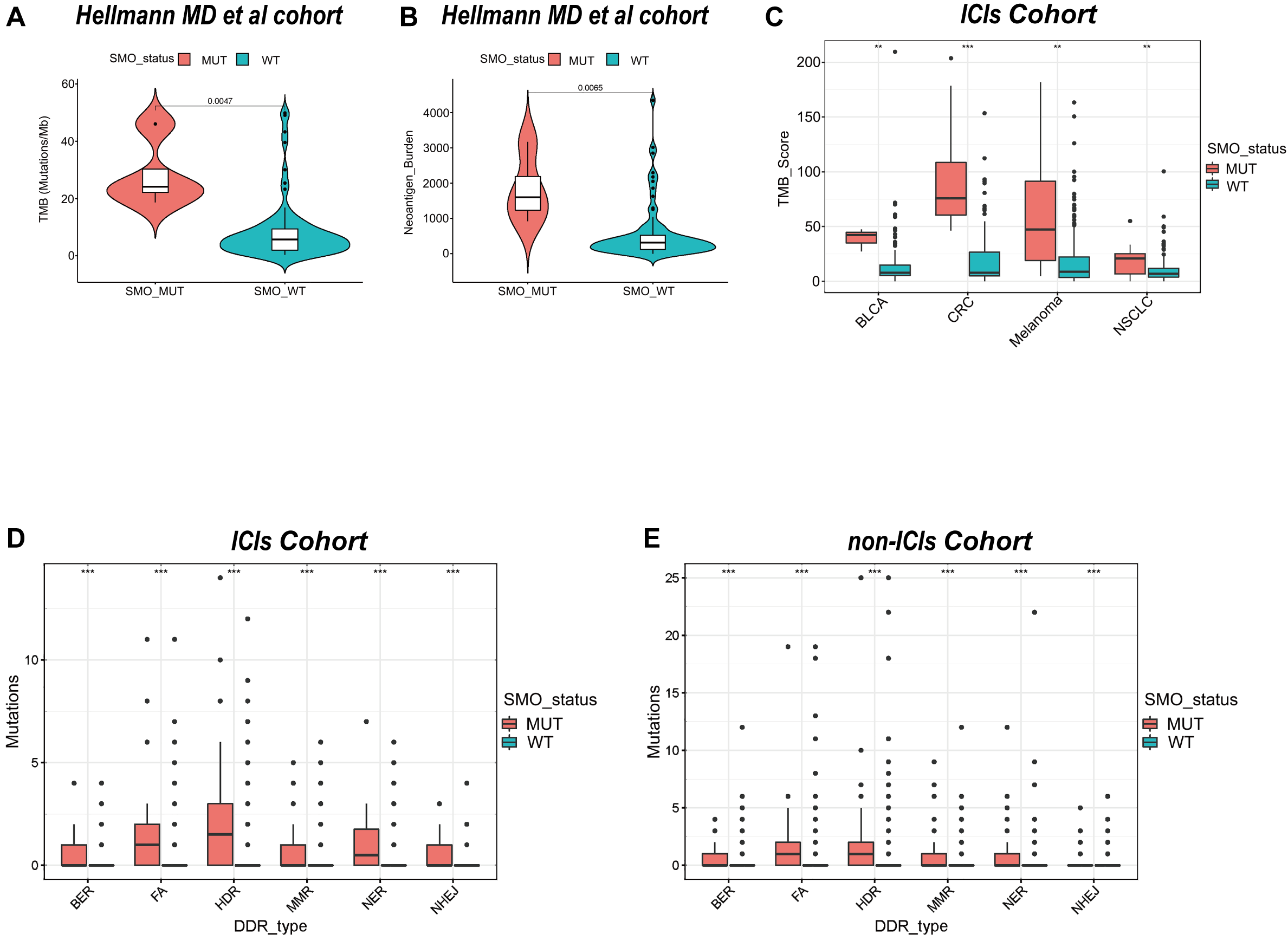

Supplement: Supplementary Figure 5 — Associations between SMO mutation status and TMB, Neoantigens load and DDR pathway gene mutation counts in the ICIs cohort and non-ICIs cohort. (A, B) Boxplot depicting the distribution of TMB, and neoantigen between SMO_MUT and SMO_WT in the Hellmann MD et al. cohort. (C) Boxplot depicting the distribution of TMB between SMO_MUT and SMO_WT in the ICIs cohort (Samstein et al). (D, E) Mutation amounts of DDR pathway gene mutation counts between SMO-mutated and wild-type subgroups in the ICIs cohort (Samstein et al) and the non-ICIs cohort (Zehir A et al). (*p < 0.05, **p <0.01, ***p <0.001, two-tailed Mann-Whitney U test). [file Image_5.tif]

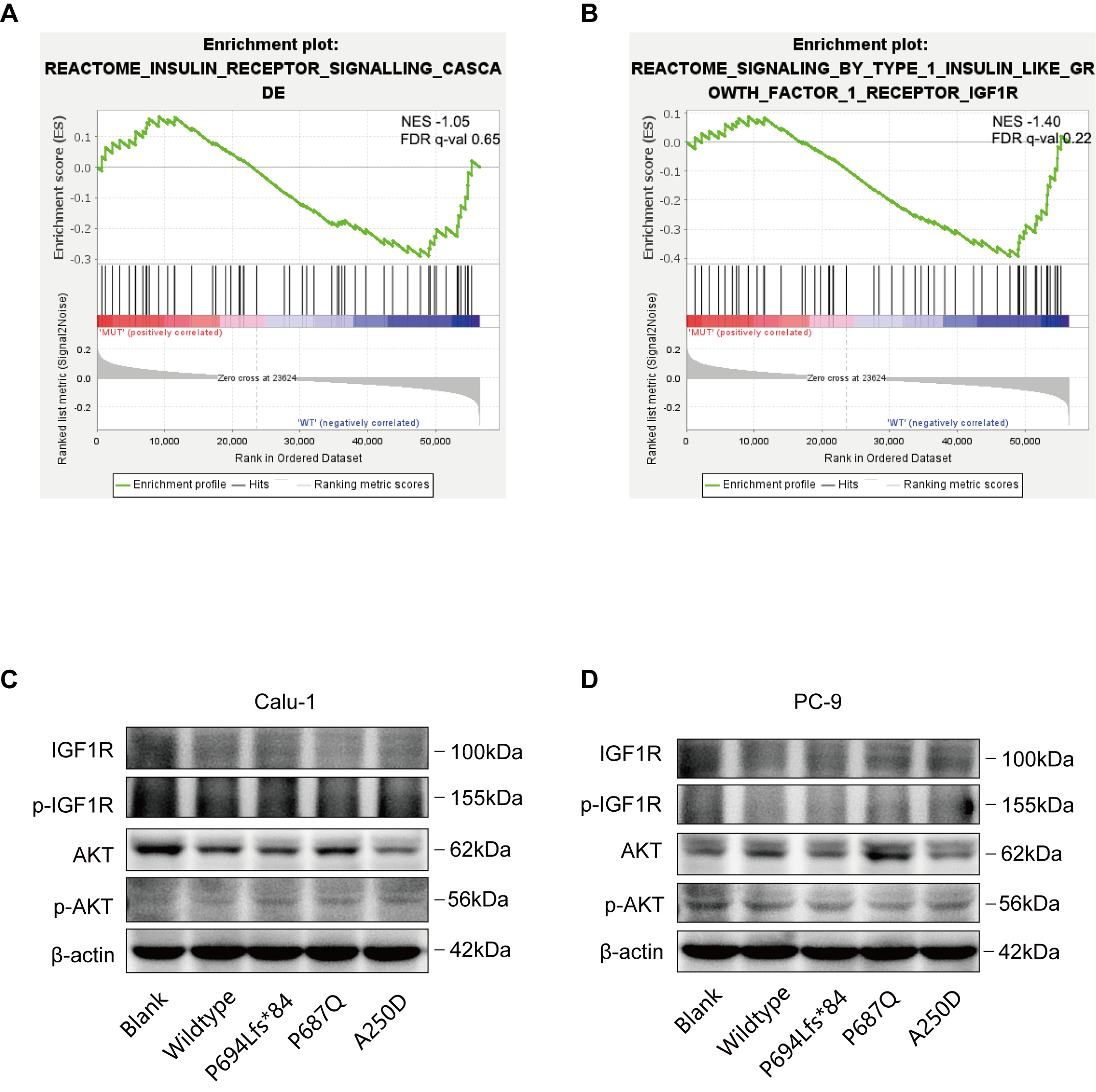

Supplement: Supplementary Figure 6 — Association between SMO mutation and IGR1R/AKT pathway. (A, B) GSEA analysis of IGF1R related signaling activity between the SMO-mutated and the wild-type tumors. (C, D) Western blot analysis of different SMO mutations on the IGF1R/AKT pathway activity. [file Image_6.tif]
