# Supplementary material for: SMO mutation predicts the effect of immune checkpoint inhibitor: From NSCLC to multiple cancers
Source: Front Immunol. 2022 Nov 3;13:955800. doi: 10.3389/fimmu.2022.955800 (PMC9669061; doi:10.3389/fimmu.2022.955800)
Supplement: Supplementary file 9 [file DataSheet_1.pdf]

Supplementary Table 1 Developmental signaling pathways-related genes from KEGG database (WNT, Notch and Hedgehog pathways).

KEGG_SIGNALING_PATHWAY	GENE_LIST					
KEGG_NOTCH_SIGNALING_PATHWAY	NOTCH4	DTX3L	NOTCH3	NOTCH2	EP300	HES1
	NUMB	PSEN2	PSEN1	PTCRA	SNW1	APH1A
	KAT2A	RFNG	CREBBP	NCOR2	RBPJ	DTX1
	DTX2	MAML1	JAG2	DVL2	NUMBL	CTBP2
	NOTCH1	DVL3	DLL4	PSENEN	DLL3	MAML2
	NCSTN	KAT2B	CIR1	DLL1	LFNG	JAG1
	DTX4	ADAM17D	CTBP1	MAML3	HDAC1	HDAC2
	MFNG	VL1	RBPJL	HES5	DTX3	
KEGG_HEDGEHOG_SIGNALING_PATHWAY	CSNK1A1L	HHIP ZIC2	PTCH2	GAS1	WNT3A	WNT9B
	BMP4	WNT9A	LRP2	CSNK1G1	WNT2B	WNT11
	PRKACGB	WNT10B	IHH	WNT10A	WNT1	CSNK1A1
	MP2	CSNK1E	SMO	WNT4	CSNK1D	STK36
	GLI2	BMP8A	CSNK1G2	CSNK1G3	RAB23	WNT5B
	BMP7	GSK3B	BTRC	SHH	WNT2	GLI1
	GLI3	WNT7A	WNT7B	WNT6	WNT3	DHH
	PTCH1	BMP5	WNT8A	FBXW11	PRKX	PRKACA
	BMP8B	BMP6	WNT8B	SUFU	WNT5A	PRKACB
	WNT16					
KEGG_WNT_SIGNALING_PATHWAY	JUN	LRP5	LRP6	DAAM2	NFAT5	SIAH1
	PPP2R1B	PPP3R2	FZD8	TBL1XR1	MYC	TBL1Y
	ROCK2	SFRP2	LEF1	MMP7	SOX17	WNT5B
	NKD1	SFRP1	CREBBP	CER1	CSNK2A1	CCND1
	FZD10	PPP3CC	FZD9	MAP3K7	CSNK2A2	CAMK2A
	FZD5	VANGL1	PRICKLE1	VANGL2	NFATC4	NLK
	NKD2	PPP3R1	CTBP2	WNT2B	CSNK1A1	CAMK2B
	TCF7L1	FZD1	ROCK1	WNT11	NFATC3	CAMK2D
	RUVBL1	FZD4	CTBP1	WNT10B	CSNK1E	CAMK2G
	PPARD	APC2	WNT9B	DKK2	BTRC	PRKACA
	PPP3CB	FZD6	WNT9A	SKP1P2	PRKX	APC
	TP53	FZD7	CTNNBIP1	CHP2	SKP1	PRKACB
	PPP3CA	SENP2	MAPK9	AXIN1	FBXW11	PRKACG
	PPP2R5A	NFATC2	MAPK10	AXIN2	RBX1	WNT16
	PPP2R5E	NFATC1	WNT3A	DKK4	CSNK2B	DAAM1
	PPP2R5D	CCND3	DVL3	WNT1	WNT5A	CHD8
	PPP2R5C	PLCB2	RAC2	MAPK8	WNT6	FRAT1
	PPP2R5B	PLCB1	DVL2	EP300	CTNNB1	CACYBP
	FZD2SFR	CSNK1A1	RAC3	WNT7A	PPP2CB	CCND2
	P5SFRP4	L	FZD3	GSK3B	PPP2CA	WNT4
	CHP1	PRKCB	DKK1	WNT7B	PPP2R1A	SMAD3
	PORCN	PLCB3	CXXC4	PSEN1	TBL1X	TCF7
	PRICKLE2	PRKCA	DVL1	WNT8A	RHOA	SMAD4
	SMAD2	PLCB4	FOSL1	WNT8B	FRAT2	RAC1
	WNT10A	WIF1	CUL1	WNT2	PRKCG	TCF7L2
	WNT3					

Supplemental Table 2 OncoScreen 68 genes list in our Lung Core panel for NGS analysis.

ALK	BRAF	EGFR	ERBB2
KRAS	MET	RET	ROS1
AKT1	APC	AR	ARAF
ATM	AXL	BCL2L11	BRCA1
BRCA2	CCND1	CD74	CDK4
CDK6	CDKN2A	CTNNB1	DDR2
ERBB3	ESR1	ERBB4	FGF19
FGF3	FGF4	FGFR1	FGFR2
FGFR3	FLT3	HRAS	IDH1
IDH2	IFG1R	JAK1	JAK2
KDR	KIT	MAP2K1	MTOR
MYC	NF1	NOTCH1	NRAS
NRG1	NTRK1	NTRK2	NTRK3
PDGFRA	PIK3CA	PTCH1	PTEN
RAF1	RB1	SMAD4	SMO
STK11	TOP2A	TP53	TSC1
TSC2	CYP2D6	DPYD	UGT1A1

Supplementary Table 3 The primer sequences for qPCR.

Primer	Forward	Reverse
SMO[1]	5′-TACCTGGTGCA GGTTGACTG-3′	5′-AGTGTGCATG CTGAAGGACA-3′
PD-L1/CD274	5′-GGCATTTGCTG AACGCATTT-3′	5′-AGGTCTTCCT CTCCATGCAC-3′
GAPDH	5′-CTGACTTCAA CAGCGACACC-3′	5′-GTGGTCCAGG GGTCTTACTC-3′

1 Primers were designed in non-frameshift mutation regions

Supplementary Table 4 Patient characteristics of the NSCLC in the NSCLC discovery cohort (database cohort) and the NSCLC validation cohort (chest hospital cohort).

	**Discovery Cohort (Database Cohort)**	**Validation Cohort (Chesthospital Cohort)**
No. of patients	349	314
**Median age, years (range)**	65 (22-92)	63 (27-85)
＞60 y	222	187
≤60y	127	127
**Gender**		
Male	171	238
Female	178	76
**Smoker**		
Ever	281	131
Never	68	163
NE	/	20
**Histology**		
Squamous	54	63
Non-squamous	295	251
**Lines of ICI treatment**		
1st line	135	133
＞1st line	314	181
**Treatment**		
PD-1/PD-L1, Monotherapy	240	/
Combination therapy	109	/
PD-1	/	139
PD-1+A[1]	/	17
PD-1+C[2]	/	158
**Best RECIST Distribution**		
CR/PR	85	72
SD	120	141
PD	137	101
NE[3]	7	/
**Dural clinical benefit**		
DCB	120	107
NDB	208	164
NE[4]	21	43
**SMO_status**		
MUT	13	11
WT	336	303
**Data source**		
Rizvi NA et al (WES)	34	/
Hellmann MD et al (part of Checkmate 012) (WES)	75	/
Rizvi H (MSK-IMPACT panel)	240	/
Chest hospital cohort	/	314

[1] PD-1+A: combination of PD-1 inhitotor and anti-vascular therapy

[2] PD-1+C: combination of PD-1 inhitotor and chemotherapy

[3]NE: Objective treatment response was recorded as not evaluable.

[4]NE: Patients who were censored before 6 months of follow-up were considered not evaluable (NE).

Supplementary Table 5 Developmental signaling pathways-related genes and corresponding clinical outcomes in the discovery cohort.

	MSK-IMPACT panel	Total cases	Proportion	CR/PR vs. PD P value[2] (adjusted P val)	DCB vs. NDB P value[3] (adjusted P val)
SMO	*included*	*349*	*3%*	*7/1(0.019)*	*9/4(0.048)*
APC	included	349	5%	7/5(0.260)	10/8(0.177)
NOTCH2	included	349	5%	4/6(1.000)	8/6(0.197)
TP53	Included	349	58%	57/70(0.049)	73/110(0.215)
CTNNB1	Included	349	4%	3/9(0.414)	2/10(0.271)
NOTCH1	included	349	6%	7/6(0.296)	9/10(0.385)
SMAD4	included	349	4%	4/7(1.000)	2/9(0.391)
CREBBP	included	349	5%	7/7(0.430)	8/10(0.521)
NOTCH3	included	349	4%	2/6(0.746)	4/9(0.813)
NOTCH4	included	349	6%	6/10(1.000)	7/12(1.000)
GLI2	not included	109	9%	5/1(0.282)	6/2(0.401)
JAG2	not included	109	3%	/	6/2(0.401)
LRP2	not included	109	8%	/	6/2(0.401)
PLCB4	not included	109	9%	/	6/2(0.401)
LRP5	not included	109	11%	/	5/5(1.000)
AXIN1	included	349	1.8%	/	5/1(0.053)
GLI1	included	294	3%	/	5/3(0.192)
AXIN2	included	349	2.4%	/	4/5(0.775)
EP300	included	349	3%	/	3/6(1.000)

[1]Target genes were confined in the genelist in TableS1.

[2]P value < 0.05 denoted the mutation significantly enrichment in CR/PR group. The gene more than 5 mutations were listed.

[3]P value < 0.05 denoted significantly enrichment in DCB group.

Abbreviation: CR, complete response; P, partial response; DCB, durable clinical benefit;

Supplementary Table 6 Univariate and multivariable Cox regression analysis of progression-free survival in the NSCLC discovery cohort.

Variable	Univariate analysis			Multivariate analysis		
	HR	95%CI	*P* value	HR	95%CI	*P* value
Histology (Sq vs. Non-Sq)	1.07	0.66-1.72	0.781	/	/	/
Gender (Male vs. Female)	0.94	0.68-1.32	0.733	/	/	/
Age (≤60y vs. ＞60y)	1.08	0.84-1.39	0.534	/	/	/
Drug type[1] (Como vs. Mono)	0.53	0.37-0.76	<0.001	0.67	0.40-1.11	0.116
Smoking history[2] (Ever vs. Never)	0.51	0.34-0.76	<0.001	0.62	0.40-0.95	0.029
Lines of ICI treatment (＞1st line vs. 1st line)	2.05	1.45-2.89	<0.001	1.63	1.00-2.66	0.052
SMO_status (MUT vs. WT)	0.15	0.04-0.61	0.008	0.21	0.05-0.86	0.030
TMB_status[3] (HIGH vs. LOW)	0.49	0.34-0.69	<0.001	0.57	0.39-0.82	0.003
PD-L1_status[4] (HIGH vs. LOW)	0.46	0.29-0.74	<0.001	0.45	0.28-0.72	<0.001

CI, confidence interval; HR, hazard ratio.

[1] **Drug type:** Mono: PD-(L)1, monotherapy; Como: PD-(L)1 + CTLA-4 combination therapy.

[2] **Ever:** including Former and current smoker.

[3] **TMB_status:** TMB_HIGH > median TMB in each cohort; TMB_LOW < median TMB in each cohort.

[4] **PD-L1_status:** PD-L1_HIGH>50% pos; PD-L1_LOW<50% pos; The sample without PD-L1 status was deleted.
