# Supplementary material for: SMO mutation predicts the effect of immune checkpoint inhibitor: From NSCLC to multiple cancers
Source: Front Immunol. 2022 Nov 3;13:955800. doi: 10.3389/fimmu.2022.955800 (PMC9669061; doi:10.3389/fimmu.2022.955800)
Supplement: Supplementary file 10 [file DataSheet_2.pdf]

Supplementary Table 7 Clinical characteristics of the NSCLC validation cohort (chest hospital cohort).

Patient_ID	Gender[1]	Age	Smoking history	Cancer_Type	NGS panel[2]	Stage	Treatment_Type[3]	lines_of_ICIs_treatment[4]	BOR[5]	DCB_status[6]	PFS(months)	PFS_status[7]	PDL1_status[8]	SMO_status[9]
Pat-001	M	53	NE	LUAD	NGS(68 gene panel)	IV	PD-1	3	SD	DCB	7.27	1	Unknown	0
Pat-002	F	73	NEVER	LUAD	NGS(68 gene panel)	IV	PD-1+C	4	PD	NDB	2.17	1	Negative(<1%)	0
Pat-003	F	72	NEVER	LUAD	NGS(68 gene panel)	IV	PD-1	4	PD	NDB	0.43	1	Unknown	0
Pat-004	M	51	NEVER	LUAD	NGS(68 gene panel)	IV	PD-1	2	PD	NDB	0.47	1	Unknown	0
Pat-005	M	67	NE	LUAD	NGS(68 gene panel)	IV	PD-1+C	4	SD	DCB	23.70	1	Negative(<1%)	0
Pat-006	F	61	NEVER	LUAD	NGS(68 gene panel)	IV	PD-1+C	3	SD	NDB	3.67	1	Unknown	0
Pat-007	M	60	EVER	LUAD	NGS(68 gene panel)	IV	PD-1	3	PD	NDB	1.10	1	Negative(<1%)	0
Pat-008	F	62	NEVER	LUAD	NGS(68 gene panel)	IV	PD-1+A	3	SD	NDB	1.63	1	Unknown	0
Pat-009	M	62	EVER	LUAD	NGS(68 gene panel)	IV	PD-1	3	SD	NDB	5.00	1	Negative(<1%)	0
Pat-010	F	66	NEVER	LUAD	NGS(68 gene panel)	IV	PD-1+C	4	PD	NDB	1.37	1	Negative(<1%)	0
Pat-011	M	59	NEVER	LUAD	NGS(68 gene panel)	IV	PD-1	2	PD	NDB	1.03	1	Unknown	0
Pat-012	F	61	NEVER	LUAD	NGS(68 gene panel)	IV	PD-1	4	PD	NDB	0.73	1	Strong(>50%)	0
Pat-013	M	77	EVER	LUAD	NGS(68 gene panel)	IV	PD-1+A	4	PD	NDB	4.87	1	Unknown	0
Pat-014	M	53	EVER	LUAD	NGS(68 gene panel)	IV	PD-1	4	SD	NDB	1.93	1	Negative(<1%)	0
Pat-015	F	52	NEVER	LUAD	NGS(68 gene panel)	IV	PD-1+C	4	PD	NDB	1.47	1	Unknown	0
Pat-016	M	57	NEVER	LUAD	NGS(68 gene panel)	IV	PD-1	3	PD	NDB	2.20	1	Weak(1-49%)	0
Pat-017	F	75	NEVER	LUAD	NGS(68 gene panel)	IV	PD-1	3	SD	NDB	5.27	1	Unknown	0
Pat-018	F	69	NEVER	LUAD	NGS(68 gene panel)	IV	PD-1	4	PD	NDB	2.13	1	Unknown	0
Pat-019	M	71	EVER	LUAD	NGS(68 gene panel)	IV	PD-1	2	SD	DCB	10.50	1	Unknown	0
Pat-020	F	64	NEVER	LUAD	NGS(68 gene panel)	IV	PD-1+C	1	SD	NE	3.73	0	Negative(<1%)	0
Pat-021	M	67	EVER	LUAD	NGS(68 gene panel)	IV	PD-1+C	1	PD	NDB	4.13	1	Negative(<1%)	0
Pat-022	F	42	NEVER	LUAD	NGS(68 gene panel)	IV	PD-1	4	SD	NDB	2.30	1	Unknown	0
Pat-023	F	57	NEVER	LUAD	NGS(68 gene panel)	IV	PD-1+C	2	SD	DCB	6.17	1	Negative(<1%)	0
Pat-024	F	57	NEVER	LUAD	NGS(68 gene panel)	IV	PD-1+C	1	SD	DCB	9.97	1	Weak(1-49%)	0
Pat-025	M	61	NEVER	LUAD	NGS(68 gene panel)	IIIB	PD-1+C	4	PD	NDB	5.13	1	Unknown	0
Pat-026	M	55	EVER	LUAD	NGS(68 gene panel)	IV	PD-1+C	4	PD	NDB	1.40	1	Unknown	0
Pat-027	M	70	EVER	LUAD	NGS(68 gene panel)	IV	PD-1+C	2	SD	DCB	9.93	1	Strong(>50%)	0
Pat-028	F	56	NEVER	LUAD	NGS(68 gene panel)	IV	PD-1	4	PD	NDB	0.43	1	Unknown	0
Pat-029	M	57	NEVER	LUAD	NGS(68 gene panel)	IV	PD-1	3	SD	NDB	5.83	1	Negative(<1%)	0
Pat-030	F	38	NEVER	LUAD	NGS(68 gene panel)	IV	PD-1+C	4	PR	NDB	5.47	1	Unknown	0
Pat-031	M	67	NEVER	LUAD	NGS(68 gene panel)	IV	PD-1	4	SD	DCB	10.47	1	Weak(1-49%)	0
Pat-032	F	69	NEVER	LUAD	NGS(68 gene panel)	IV	PD-1	2	SD	NDB	1.83	1	Unknown	0
Pat-033	M	60	NEVER	LUAD	NGS(68 gene panel)	IV	PD-1	2	PD	NDB	1.57	1	Unknown	0
Pat-034	F	60	NEVER	LUAD	NGS(68 gene panel)	IV	PD-1	4	PD	NDB	1.57	1	Weak(1-49%)	0
Pat-035	F	43	NEVER	LUAD	NGS(68 gene panel)	IV	PD-1+C	4	SD	NDB	5.33	1	Negative(<1%)	0
Pat-036	F	61	NEVER	LUAD	NGS(68 gene panel)	IV	PD-1	4	PD	NDB	0.93	1	Unknown	0
Pat-037	M	61	EVER	LUAD	NGS(68 gene panel)	IV	PD-1	3	PD	NDB	0.87	1	Negative(<1%)	0
Pat-038	F	68	NEVER	LUAD	NGS(68 gene panel)	IV	PD-1	4	PD	NDB	0.93	1	Unknown	0
Pat-039	M	67	EVER	LUAD	NGS(68 gene panel)	IV	PD-1	3	SD	DCB	17.30	1	Weak(1-49%)	0
Pat-040	F	38	NEVER	LUAD	NGS(68 gene panel)	IV	PD-1	4	PD	NDB	0.83	1	Unknown	0
Pat-041	F	66	NEVER	LUAD	NGS(68 gene panel)	IV	PD-1	2	SD	NE	1.90	0	Unknown	0
Pat-042	M	56	EVER	LUAD	NGS(68 gene panel)	IV	PD-1+A	4	SD	NDB	3.03	1	Unknown	0
Pat-043	M	63	EVER	LUAD	NGS(68 gene panel)	IV	PD-1	2	PR	DCB	6.77	0	Negative(<1%)	0
Pat-044	F	70	NEVER	LUAD	NGS(68 gene panel)	IV	PD-1	2	PD	NDB	1.73	1	Unknown	0
Pat-045	M	57	EVER	LUAD	NGS(68 gene panel)	IV	PD-1+C	1	PR	NE	4.90	0	Unknown	0
Pat-046	M	63	EVER	LUAD	NGS(68 gene panel)	IV	PD-1	3	SD	NE	1.67	0	Unknown	0
Pat-047	F	44	NEVER	LUAD	NGS(68 gene panel)	IV	PD-1+C	2	SD	NDB	5.43	1	Unknown	0
Pat-048	F	52	NEVER	LUAD	NGS(68 gene panel)	IV	PD-1+C	1	SD	DCB	6.07	1	Negative(<1%)	0
Pat-049	F	52	NEVER	LUAD	NGS(68 gene panel)	IV	PD-1+C	3	PD	NDB	2.07	1	Strong(>50%)	0
Pat-050	M	66	EVER	LUAD	NGS(68 gene panel)	IIIB	PD-1	2	PD	NDB	0.67	1	Weak(1-49%)	0
Pat-051	F	57	NEVER	LUAD	NGS(68 gene panel)	IV	PD-1	2	PD	NDB	1.83	1	Unknown	0
Pat-052	F	65	EVER	LUAD	NGS(68 gene panel)	IV	PD-1	1	PD	NDB	1.43	1	Unknown	0
Pat-053	M	38	NEVER	LUAD	NGS(68 gene panel)	IV	PD-1+C	4	SD	NE	1.83	0	Negative(<1%)	0
Pat-054	F	51	NEVER	LUAD	NGS(68 gene panel)	IV	PD-1	2	PD	NDB	1.00	1	Weak(1-49%)	0
Pat-055	M	72	NE	LUAD	NGS(68 gene panel)	IV	PD-1+A	4	SD	DCB	7.60	0	Weak(1-49%)	Q380* (11.97%)
Pat-056	M	64	EVER	LUAD	NGS(68 gene panel)	IV	PD-1	2	SD	NDB	5.93	1	Unknown	0
Pat-057	F	55	NEVER	LUAD	NGS(68 gene panel)	IV	PD-1+A	3	SD	DCB	12.13	1	Unknown	0
Pat-058	M	65	EVER	LUAD	NGS(68 gene panel)	IV	PD-1	2	SD	NDB	2.57	1	Unknown	0
Pat-059	M	37	EVER	LUAD	NGS(68 gene panel)	IV	PD-1+C	2	SD	NDB	2.37	1	Negative(<1%)	0
Pat-060	M	70	NEVER	LUAD	NGS(68 gene panel)	IV	PD-1	3	SD	DCB	24.80	0	Strong(>50%)	G422V (20.22%)
Pat-061	F	57	NEVER	LUAD	NGS(68 gene panel)	IV	PD-1+A	4	PD	NDB	0.77	1	Unknown	0
Pat-062	M	68	EVER	LUAD	NGS(68 gene panel)	IV	PD-1	4	PD	NDB	1.70	1	Strong(>50%)	0
Pat-063	M	56	NEVER	LUAD	NGS(68 gene panel)	IV	PD-1+C	2	PR	DCB	6.50	0	Unknown	0
Pat-064	M	56	EVER	LUAD	NGS(68 gene panel)	IV	PD-1	1	SD	DCB	21.60	1	Strong(>50%)	0
Pat-065	F	67	NEVER	LUAD	NGS(68 gene panel)	IV	PD-1+C	4	SD	DCB	6.93	1	Unknown	0
Pat-066	M	70	EVER	LUAD	NGS(68 gene panel)	IIIB	PD-1	2	SD	DCB	6.30	1	Weak(1-49%)	0
Pat-067	M	63	EVER	LUAD	NGS(68 gene panel)	IV	PD-1	2	PD	NDB	3.77	1	Weak(1-49%)	0
Pat-068	M	46	NE	LUAD	NGS(68 gene panel)	IV	PD-1+C	3	SD	DCB	7.10	0	Unknown	0
Pat-069	M	61	EVER	LUAD	NGS(68 gene panel)	IIIB	PD-1	3	SD	DCB	7.60	1	Strong(>50%)	0
Pat-070	F	59	NEVER	LUAD	NGS(68 gene panel)	IV	PD-1+C	3	SD	NDB	5.20	1	Negative(<1%)	0
Pat-071	M	60	EVER	LUAD	NGS(68 gene panel)	IV	PD-1+C	3	SD	NDB	5.83	1	Unknown	N309I (28.75%)
Pat-072	M	68	EVER	LUAD	NGS(68 gene panel)	IV	PD-1+C	3	SD	NDB	3.60	1	Unknown	0
Pat-073	M	54	NEVER	LUAD	NGS(68 gene panel)	IV	PD-1+C	3	PD	NDB	1.80	1	Unknown	0
Pat-074	M	64	EVER	LUAD	NGS(68 gene panel)	IV	PD-1	2	PD	NDB	0.27	1	Unknown	0
Pat-075	M	70	EVER	LUAD	NGS(68 gene panel)	IV	PD-1	3	SD	NDB	2.27	1	Unknown	0
Pat-076	F	63	NEVER	LUAD	NGS(68 gene panel)	IV	PD-1+C	4	SD	DCB	6.53	0	Negative(<1%)	0
Pat-077	M	78	NEVER	LUAD	NGS(68 gene panel)	IV	PD-1	2	PD	NDB	0.97	1	Unknown	0
Pat-078	M	53	EVER	LUAD	NGS(68 gene panel)	IV	PD-1	2	SD	DCB	11.53	1	Negative(<1%)	0
Pat-079	F	55	NEVER	LUAD	NGS(68 gene panel)	IV	PD-1	2	PD	NDB	0.50	1	Negative(<1%)	0

Continued on following page

Patient_ID	Gender[1]	Age	Smoking history	Cancer _Type	NGS panel[2]	Stage	Treatment_Type[3]	lines_of _ICIs_treatment[4]	BOR[5]	DCB_status[6]	PFS(months)	PFS_status[7]	PDL1_status[8]	SMO_status[9]
Pat-080	M	50	EVER	LUAD	NGS(68 gene panel)	IV	PD-1	3	PD	NDB	1.67	1	Unknown	0
Pat-081	F	80	NEVER	LUAD	NGS(68 gene panel)	IV	PD-1	2	PD	NDB	0.93	1	Weak(1-49%)	0
Pat-082	M	44	EVER	LUAD	NGS(68 gene panel)	IV	PD-1	3	SD	NDB	1.90	1	Unknown	0
Pat-083	M	54	NE	LUAD	NGS(68 gene panel)	IV	PD-1	4	PD	NDB	0.37	1	Strong(>50%)	0
Pat-084	F	69	NEVER	LUAD	NGS(68 gene panel)	IV	PD-1	4	PD	NDB	1.13	1	Negative(<1%)	0
Pat-085	M	68	NE	LUAD	NGS(68 gene panel)	IV	PD-1+C	3	SD	DCB	9.13	1	Unknown	0
Pat-086	F	69	NEVER	LUAD	NGS(68 gene panel)	IV	PD-1	2	SD	DCB	6.03	1	Negative(<1%)	0
Pat-087	M	46	EVER	LUAD	NGS(68 gene panel)	IV	PD-1+A	4	PD	NDB	0.93	1	Negative(<1%)	0
Pat-088	F	65	NEVER	LUAD	NGS(68 gene panel)	IV	PD-1+C	2	SD	NDB	4.83	1	Unknown	0
Pat-089	M	70	EVER	LUAD	NGS(68 gene panel)	IV	PD-1	2	SD	DCB	6.37	0	Strong(>50%)	0
Pat-090	F	66	NEVER	LUAD	NGS(68 gene panel)	IV	PD-1+C	2	SD	NDB	2.23	1	Weak(1-49%)	0
Pat-091	M	54	NEVER	LUAD	NGS(68 gene panel)	IV	PD-1	2	PD	NDB	0.40	1	Unknown	0
Pat-092	F	67	EVER	LUAD	NGS(68 gene panel)	IV	PD-1	3	PD	NDB	0.47	1	Unknown	0
Pat-093	F	59	NEVER	LUAD	NGS(68 gene panel)	IV	PD-1	2	PD	NDB	1.43	1	Negative(<1%)	0
Pat-094	M	73	NEVER	LUAD	NGS(68 gene panel)	IV	PD-1+C	2	SD	DCB	7.87	1	Negative(<1%)	0
Pat-095	F	70	NEVER	LUAD	NGS(68 gene panel)	IV	PD-1	2	SD	NE	1.00	0	Negative(<1%)	0
Pat-096	M	66	NEVER	LUAD	NGS(68 gene panel)	IV	PD-1+C	2	SD	NDB	4.77	1	Unknown	0
Pat-097	F	27	NEVER	LUAD	NGS(68 gene panel)	IV	PD-1+A	4	PD	NDB	2.07	1	Strong(>50%)	0
Pat-098	F	38	NEVER	LUAD	NGS(68 gene panel)	IV	PD-1	2	SD	NDB	2.00	1	Strong(>50%)	0
Pat-099	M	51	EVER	LUAD	NGS(68 gene panel)	IV	PD-1	2	PR	DCB	7.30	1	Weak(1-49%)	0
Pat-100	M	66	NEVER	LUAD	NGS(68 gene panel)	IV	PD-1+C	1	SD	NE	5.10	0	Unknown	0
Pat-101	M	51	EVER	LUAD	NGS(68 gene panel)	IV	PD-1	3	PD	NDB	1.73	1	Unknown	0
Pat-102	M	72	EVER	LUAD	NGS(68 gene panel)	IV	PD-1+C	4	PD	NDB	3.47	1	Unknown	0
Pat-103	M	56	EVER	LUAD	NGS(68 gene panel)	IV	PD-1	2	SD	NE	5.33	0	Strong(>50%)	0
Pat-104	F	70	NEVER	LUAD	NGS(68 gene panel)	IV	PD-1+C	2	PR	DCB	22.57	0	Strong(>50%)	0
Pat-105	M	58	EVER	LUAD	NGS(68 gene panel)	IV	PD-1+C	1	PR	DCB	29.60	0	Negative(<1%)	0
Pat-106	M	64	EVER	LUAD	NGS(68 gene panel)	IV	PD-1	3	PD	NDB	1.80	1	Unknown	0
Pat-107	M	52	EVER	LUAD	NGS(68 gene panel)	IV	PD-1+C	1	PR	DCB	12.30	1	Unknown	0
Pat-108	F	57	NEVER	LUAD	NGS(68 gene panel)	IV	PD-1+A	2	SD	NDB	2.57	1	Weak(1-49%)	0
Pat-109	M	72	NEVER	LUAD	NGS(68 gene panel)	IIIB	PD-1+C	4	PD	NDB	0.70	1	Negative(<1%)	0
Pat-110	M	56	NEVER	LUAD	NGS(68 gene panel)	IV	PD-1	4	SD	NDB	2.57	1	Negative(<1%)	0
Pat-111	F	60	NEVER	LUAD	NGS(68 gene panel)	IIIB	PD-1+C	1	SD	DCB	13.77	1	Unknown	0
Pat-112	M	53	NEVER	LUAD	NGS(68 gene panel)	IV	PD-1+C	1	SD	DCB	10.30	1	Negative(<1%)	0
Pat-113	M	54	EVER	LUAD	NGS(68 gene panel)	IV	PD-1+C	2	PR	NDB	5.57	1	Negative(<1%)	0
Pat-114	F	66	NEVER	LUAD	NGS(68 gene panel)	IV	PD-1+C	1	PD	NDB	1.70	1	Unknown	0
Pat-115	M	68	EVER	LUAD	NGS(68 gene panel)	IV	PD-1	3	PD	NDB	0.90	1	Unknown	0
Pat-116	M	61	EVER	LUAD	NGS(68 gene panel)	IV	PD-1	2	SD	NDB	1.83	1	Unknown	0
Pat-117	M	64	EVER	LUAD	NGS(68 gene panel)	IV	PD-1+C	1	SD	NDB	4.40	1	Strong(>50%)	0
Pat-118	F	65	NEVER	LUAD	NGS(68 gene panel)	IV	PD-1+C	1	SD	DCB	8.53	1	Unknown	0
Pat-119	M	64	EVER	LUAD	NGS(68 gene panel)	IV	PD-1+C	1	PD	NDB	0.67	1	Unknown	0
Pat-120	M	74	NEVER	LUAD	NGS(68 gene panel)	IIIB	PD-1	4	SD	NE	1.97	0	Unknown	0
Pat-121	F	63	NEVER	LUAD	NGS(68 gene panel)	IV	PD-1+A	4	SD	NDB	4.70	1	Negative(<1%)	0
Pat-122	M	68	EVER	LUAD	NGS(68 gene panel)	IV	PD-1+C	1	PR	DCB	27.20	0	Strong(>50%)	0
Pat-123	M	57	NEVER	LUAD	NGS(68 gene panel)	IV	PD-1+C	1	SD	DCB	20.30	0	Unknown	0
Pat-124	M	72	EVER	LUAD	NGS(68 gene panel)	IV	PD-1+C	2	PD	NDB	0.73	1	Negative(<1%)	0
Pat-125	M	39	EVER	LUAD	NGS(68 gene panel)	IV	PD-1	4	PD	NDB	0.67	1	Negative(<1%)	0
Pat-126	F	67	NEVER	LUAD	NGS(68 gene panel)	IIIB	PD-1+A	4	PD	NDB	2.27	1	Strong(>50%)	0
Pat-127	M	68	NE	LUAD	NGS(68 gene panel)	IV	PD-1+C	1	PR	DCB	12.47	0	Unknown	0
Pat-128	M	41	EVER	LUAD	NGS(68 gene panel)	IV	PD-1+C	2	SD	DCB	7.27	1	Weak(1-49%)	0
Pat-129	F	60	NEVER	LUAD	NGS(68 gene panel)	IV	PD-1+C	1	PR	NDB	4.63	1	Weak(1-49%)	0
Pat-130	M	56	NEVER	LUAD	NGS(68 gene panel)	IV	PD-1+C	1	PR	DCB	9.30	1	Weak(1-49%)	L426M（17.3%）
Pat-131	M	56	NEVER	LUAD	NGS(68 gene panel)	IIIB	PD-1+C	1	PR	DCB	6.23	1	Weak(1-49%)	Y75N(8.71%)
Pat-132	M	79	EVER	LUAD	NGS(68 gene panel)	IV	PD-1	2	PD	NDB	1.30	1	Negative(<1%)	0
Pat-133	M	57	EVER	LUAD	NGS(68 gene panel)	IV	PD-1+C	1	PD	NDB	3.27	1	Weak(1-49%)	0
Pat-134	M	51	NEVER	LUAD	NGS(68 gene panel)	IV	PD-1	3	SD	NE	5.63	0	Unknown	0
Pat-135	M	56	EVER	LUAD	NGS(68 gene panel)	IV	PD-1+C	1	SD	DCB	6.67	0	Strong(>50%)	0
Pat-136	M	62	NEVER	LUAD	NGS(68 gene panel)	IV	PD-1	2	PD	NDB	0.77	1	Unknown	0
Pat-137	M	74	EVER	LUAD	NGS(68 gene panel)	IIIB	PD-1	2	SD	DCB	8.93	1	Negative(<1%)	0
Pat-138	M	85	NEVER	LUAD	NGS(68 gene panel)	IIIB	PD-1	1	PD	NDB	1.57	1	Weak(1-49%)	0
Pat-139	M	70	EVER	LUAD	NGS(68 gene panel)	IV	PD-1+C	1	SD	NE	4.93	0	Negative(<1%)	0
Pat-140	M	70	EVER	LUAD	NGS(68 gene panel)	IV	PD-1	2	PR	DCB	12.10	0	Unknown	0
Pat-141	M	71	EVER	LUAD	NGS(68 gene panel)	IIIB	PD-1	2	PD	NDB	0.43	1	Weak(1-49%)	0
Pat-142	M	71	NEVER	LUAD	NGS(68 gene panel)	IV	PD-1	4	PD	NDB	0.70	1	Unknown	0
Pat-143	F	44	EVER	LUAD	NGS(68 gene panel)	IV	PD-1+C	1	SD	DCB	9.07	1	Negative(<1%)	0
Pat-144	M	53	NEVER	LUAD	NGS(68 gene panel)	IV	PD-1+C	1	SD	NDB	2.00	1	Strong(>50%)	0
Pat-145	M	62	NEVER	LUAD	NGS(68 gene panel)	IV	PD-1+C	1	PD	NDB	0.33	1	Unknown	0
Pat-146	M	60	NEVER	LUAD	NGS(68 gene panel)	IIIB	PD-1+C	1	SD	NDB	2.23	1	Negative(<1%)	0
Pat-147	M	66	EVER	LUAD	NGS(68 gene panel)	IV	PD-1	2	SD	DCB	7.10	1	Weak(1-49%)	0
Pat-148	M	50	EVER	LUAD	NGS(68 gene panel)	IV	PD-1+C	1	SD	DCB	20.33	1	Weak(1-49%)	0
Pat-149	M	63	NEVER	LUAD	NGS(68 gene panel)	IV	PD-1+A	4	PD	NDB	0.90	1	Weak(1-49%)	0
Pat-150	F	57	EVER	LUAD	NGS(68 gene panel)	IV	PD-1	2	PD	NDB	1.77	1	Weak(1-49%)	0
Pat-151	F	43	NEVER	LUAD	NGS(68 gene panel)	IV	PD-1+C	2	SD	NDB	5.53	1	Strong(>50%)	0
Pat-152	M	55	EVER	LUAD	NGS(68 gene panel)	IV	PD-1+C	1	SD	NDB	2.63	1	Negative(<1%)	0
Pat-153	M	58	EVER	LUAD	NGS(68 gene panel)	IV	PD-1+C	1	PR	DCB	20.07	0	Strong(>50%)	0
Pat-154	M	63	EVER	LUAD	NGS(68 gene panel)	IIIB	PD-1	1	SD	DCB	12.17	1	Unknown	0
Pat-155	M	62	EVER	LUAD	NGS(68 gene panel)	IV	PD-1+C	3	PR	DCB	12.43	1	Negative(<1%)	0
Pat-156	M	37	NEVER	LUAD	NGS(68 gene panel)	IV	PD-1+C	4	PD	NDB	1.37	1	Weak(1-49%)	0
Pat-157	F	43	EVER	LUAD	NGS(68 gene panel)	IV	PD-1+C	1	PD	NDB	5.97	1	Negative(<1%)	0
Pat-158	M	52	EVER	LUAD	NGS(68 gene panel)	IV	PD-1+C	1	SD	NDB	5.33	1	Weak(1-49%)	0
Pat-159	M	60	EVER	LUAD	NGS(68 gene panel)	IV	PD-1	2	SD	NDB	3.27	1	Negative(<1%)	0
Pat-160	M	67	NEVER	LUAD	NGS(68 gene panel)	IV	PD-1+C	1	SD	NDB	2.57	1	Unknown	0

Continued on following page

Patient_ID	Gender[1]	Age	Smoking history	Cancer_Type	NGS panel[2]	Stage	Treatment_Type[3]	lines_of_ICIs_treatment[4]	BOR[5]	DCB_status[6]	PFS(months)	PFS_status[7]	PDL1_status[8]	SMO_status[9]
Pat-161	M	55	NEVER	LUAD	NGS(68 gene panel)	IV	PD-1+C	3	PD	NDB	0.90	1	Weak(1-49%)	0
Pat-162	M	55	EVER	LUAD	NGS(68 gene panel)	IV	PD-1+C	4	SD	NDB	0.63	1	Negative(<1%)	0
Pat-163	M	65	EVER	LUAD	NGS(68 gene panel)	IV	PD-1+C	1	SD	NE	2.33	0	Weak(1-49%)	0
Pat-164	M	64	EVER	LUAD	NGS(68 gene panel)	IV	PD-1	1	SD	DCB	18.07	1	Strong(>50%)	0
Pat-165	M	60	NEVER	LUAD	NGS(68 gene panel)	IV	PD-1+C	1	PR	DCB	6.33	1	Strong(>50%)	0
Pat-166	F	57	NEVER	LUAD	NGS(68 gene panel)	IV	PD-1+C	1	PR	DCB	11.90	1	Weak(1-49%)	0
Pat-167	F	54	NEVER	LUAD	NGS(68 gene panel)	IV	PD-1+C	1	PD	NDB	0.63	1	Unknown	0
Pat-168	M	56	NE	LUAD	NGS(68 gene panel)	IV	PD-1+A	2	SD	NE	2.10	0	Unknown	0
Pat-169	F	68	NEVER	LUAD	NGS(68 gene panel)	IV	PD-1+C	1	PR	NE	2.80	0	Negative(<1%)	0
Pat-170	F	68	NEVER	LUAD	NGS(68 gene panel)	IV	PD-1+C	2	SD	NDB	2.83	1	Weak(1-49%)	0
Pat-171	M	45	EVER	LUAD	NGS(68 gene panel)	IV	PD-1+C	1	SD	NDB	1.90	1	Strong(>50%)	0
Pat-172	M	68	NEVER	LUAD	NGS(68 gene panel)	IV	PD-1+C	1	SD	DCB	17.87	1	Weak(1-49%)	0
Pat-173	F	60	NEVER	LUAD	NGS(68 gene panel)	IV	PD-1+C	1	PR	DCB	18.07	0	Strong(>50%)	0
Pat-174	F	64	EVER	LUAD	NGS(68 gene panel)		PD-1	2	SD	DCB	7.90	1	Weak(1-49%)	0
Pat-175	M	66	NE	LUAD	NGS(68 gene panel)	IV	PD-1	2	SD	DCB	7.13	1	Strong(>50%)	0
Pat-176	M	67	NEVER	LUAD	NGS(68 gene panel)	IV	PD-1+C	1	PR	DCB	6.30	1	Negative(<1%)	0
Pat-177	M	62	NEVER	LUAD	NGS(68 gene panel)	IV	PD-1+C	4	PD	NDB	0.43	1	Weak(1-49%)	0
Pat-178	F	71	EVER	LUAD	NGS(68 gene panel)	IV	PD-1+C	1	SD	DCB	14.30	1	Weak(1-49%)	0
Pat-179	M	37	EVER	LUAD	NGS(68 gene panel)	IIIB	PD-1+C	1	SD	DCB	8.10	1	Negative(<1%)	0
Pat-180	M	66	EVER	LUAD	NGS(68 gene panel)	IV	PD-1	2	PD	NDB	0.67	1	Unknown	0
Pat-181	M	69	NEVER	LUAD	NGS(68 gene panel)	IV	PD-1	3	PD	NDB	0.53	1	Negative(<1%)	0
Pat-182	M	58	NE	LUAD	NGS(68 gene panel)	IV	PD-1	2	SD	DCB	6.83	1	Unknown	0
Pat-183	M	65	EVER	LUAD	NGS(68 gene panel)	IV	PD-1	2	PD	NDB	1.00	1	Strong(>50%)	0
Pat-184	M	65	NEVER	LUAD	NGS(68 gene panel)	IV	PD-1	2	PR	DCB	7.10	0	Strong(>50%)	0
Pat-185	M	69	NEVER	LUAD	NGS(68 gene panel)	IV	PD-1	2	PD	NDB	0.90	1	Negative(<1%)	0
Pat-186	M	67	EVER	LUAD	NGS(68 gene panel)	IV	PD-1	2	PR	NDB	3.63	1	Strong(>50%)	0
Pat-187	M	70	NEVER	LUAD	NGS(68 gene panel)	IV	PD-1	2	SD	DCB	7.27	0	Weak(1-49%)	0
Pat-188	M	68	EVER	LUAD	NGS(68 gene panel)	IV	PD-1+C	1	PR	DCB	12.83	0	Weak(1-49%)	0
Pat-189	F	63	EVER	LUAD	NGS(68 gene panel)	IV	PD-1	1	PD	NDB	0.23	1	Strong(>50%)	0
Pat-190	M	65	EVER	LUAD	NGS(68 gene panel)	IV	PD-1+C	1	PR	NE	2.07	0	Weak(1-49%)	0
Pat-191	M	70	EVER	LUAD	NGS(68 gene panel)	IV	PD-1+C	1	PR	NE	3.33	0	Weak(1-49%)	A30* (4.91%)
Pat-192	M	69	EVER	LUAD	NGS(68 gene panel)	IV	PD-1	2	SD	DCB	7.03	0	Strong(>50%)	0
Pat-193	M	68	EVER	LUAD	NGS(68 gene panel)	IV	PD-1	2	PD	NDB	1.20	1	Negative(<1%)	0
Pat-194	M	62	EVER	LUAD	NGS(68 gene panel)	IV	PD-1+A	4	SD	DCB	9.20	1	Negative(<1%)	0
Pat-195	M	72	NE	LUAD	NGS(68 gene panel)	IIIB	PD-1	1	SD	NDB	4.30	1	Strong(>50%)	0
Pat-196	M	58	EVER	LUAD	NGS(68 gene panel)	IV	PD-1	1	SD	DCB	6.13	1	Strong(>50%)	0
Pat-197	M	58	EVER	LUAD	NGS(68 gene panel)	IV	PD-1+C	1	PR	DCB	11.00	1	Strong(>50%)	0
Pat-198	M	61	NEVER	LUAD	NGS(68 gene panel)	IIIB	PD-1+C	1	PR	DCB	15.90	1	Weak(1-49%)	0
Pat-199	F	66	NEVER	LUAD	NGS(68 gene panel)	IV	PD-1	2	SD	DCB	6.57	1	Strong(>50%)	0
Pat-200	M	31	EVER	LUAD	NGS(68 gene panel)	IV	PD-1+C	1	SD	DCB	11.43	1	Unknown	0
Pat-201	M	70	EVER	LUAD	NGS(68 gene panel)	IV	PD-1+C	1	PR	NE	4.23	0	Unknown	0
Pat-202	M	63	NEVER	LUAD	NGS(68 gene panel)	IV	PD-1+C	1	PR	DCB	9.53	0	Negative(<1%)	0
Pat-203	M	47	EVER	LUAD	NGS(68 gene panel)	IV	PD-1+C	4	SD	NDB	5.33	1	Negative(<1%)	0
Pat-204	M	71	NEVER	LUAD	NGS(68 gene panel)	IV	PD-1+C	2	PD	NDB	3.30	1	Negative(<1%)	0
Pat-205	F	50	NEVER	LUAD	NGS(68 gene panel)	IV	PD-1+C	1	SD	DCB	6.90	0	Unknown	0
Pat-206	M	78	NEVER	LUAD	NGS(68 gene panel)	IV	PD-1	1	PR	DCB	9.10	0	Strong(>50%)	0
Pat-207	F	49	EVER	LUAD	NGS(68 gene panel)	IV	PD-1+C	1	PR	DCB	9.93	0	Strong(>50%)	0
Pat-208	M	64	EVER	LUAD	NGS(68 gene panel)	IIIB	PD-1+C	1	SD	DCB	7.60	1	Weak(1-49%)	0
Pat-209	M	73	NEVER	LUAD	NGS(68 gene panel)	IV	PD-1+C	1	PR	NE	1.40	0	Strong(>50%)	0
Pat-210	F	38	EVER	LUAD	NGS(68 gene panel)	IV	PD-1+C	1	PD	NDB	0.53	1	Unknown	0
Pat-211	M	56	EVER	LUAD	NGS(68 gene panel)	IIIB	PD-1+C	1	PR	DCB	9.50	0	Unknown	0
Pat-212	M	68	NEVER	LUAD	NGS(68 gene panel)	IV	PD-1+C	1	PR	DCB	7.57	1	Weak(1-49%)	0
Pat-213	M	76	NEVER	LUAD	NGS(68 gene panel)	IV	PD-1+C	1	SD	DCB	7.43	0	Negative(<1%)	0
Pat-214	M	52	EVER	LUAD	NGS(68 gene panel)	IV	PD-1+C	1	PR	NDB	4.93	1	Strong(>50%)	0
Pat-215	M	67	EVER	LUAD	NGS(68 gene panel)	IV	PD-1	2	SD	NDB	1.63	1	Negative(<1%)	0
Pat-216	M	70	EVER	LUAD	NGS(68 gene panel)	IIIB	PD-1+C	1	SD	DCB	7.40	1	Negative(<1%)	0
Pat-217	M	79	NE	LUAD	NGS(68 gene panel)	IV	PD-1	1	PR	NE	4.17	0	Strong(>50%)	0
Pat-218	M	60	NEVER	LUAD	NGS(68 gene panel)	IV	PD-1+C	1	PD	NDB	2.80	1	Negative(<1%)	0
Pat-219	F	58	NEVER	LUAD	NGS(68 gene panel)	IV	PD-1+C	1	PR	DCB	6.03	0	Strong(>50%)	0
Pat-220	F	37	EVER	LUAD	NGS(68 gene panel)	IV	PD-1+C	1	PR	DCB	19.07	1	Strong(>50%)	0
Pat-221	M	62	EVER	LUAD	NGS(68 gene panel)	IV	PD-1	2	PD	NDB	2.37	1	Negative(<1%)	0
Pat-222	M	61	EVER	LUAD	NGS(68 gene panel)	IIIB	PD-1	2	PD	NDB	1.43	1	Weak(1-49%)	0
Pat-223	M	79	EVER	LUAD	NGS(68 gene panel)	IV	PD-1	1	PR	NE	5.37	0	Weak(1-49%)	0
Pat-224	M	52	EVER	LUAD	NGS(68 gene panel)	IV	PD-1	1	PD	NDB	0.47	1	Strong(>50%)	0
Pat-225	M	73	EVER	LUAD	NGS(68 gene panel)	IV	PD-1+C	1	PR	DCB	7.23	1	Strong(>50%)	0
Pat-226	M	66	EVER	LUAD	NGS(68 gene panel)	IV	PD-1	1	PR	NE	4.97	0	Strong(>50%)	0
Pat-227	M	59	NEVER	LUAD	NGS(68 gene panel)	IV	PD-1+C	1	SD	NE	1.63	0	Unknown	0
Pat-228	F	64	EVER	LUAD	NGS(68 gene panel)	IV	PD-1+C	2	PD	NDB	3.47	1	Negative(<1%)	0
Pat-229	M	58	NEVER	LUAD	NGS(68 gene panel)	IV	PD-1+C	4	PR	NDB	4.67	1	Strong(>50%)	0
Pat-230	M	69	NEVER	LUAD	NGS(68 gene panel)	IV	PD-1	1	PR	NE	3.93	0	Strong(>50%)	0
Pat-231	M	66	EVER	LUAD	NGS(68 gene panel)	IIIB	PD-1	1	PD	NDB	1.10	1	Strong(>50%)	0
Pat-232	M	58	NEVER	LUAD	NGS(68 gene panel)	IV	PD-1+C	1	PR	NE	2.97	0	Weak(1-49%)	0
Pat-233	M	63	EVER	LUAD	NGS(68 gene panel)	IIIB	PD-1+C	1	SD	NE	3.63	0	Strong(>50%)	0
Pat-234	M	64	EVER	LUAD	NGS(68 gene panel)	IV	PD-1+C	1	PD	NDB	0.93	1	Strong(>50%)	0
Pat-235	F	46	EVER	LUAD	NGS(68 gene panel)	IIIB	PD-1+C	1	SD	NDB	4.10	1	Strong(>50%)	0
Pat-236	M	64	NEVER	LUAD	NGS(68 gene panel)	IV	PD-1+C	1	PD	NDB	1.53	1	Negative(<1%)	0
Pat-237	M	48	NEVER	LUAD	NGS(68 gene panel)	IV	PD-1+C	1	SD	NE	1.47	0	Negative(<1%)	0
Pat-238	F	42	NEVER	LUAD	NGS(68 gene panel)	IV	PD-1+C	1	PR	NE	1.53	0	Negative(<1%)	0
Pat-239	M	56	EVER	LUAD	NGS(68 gene panel)	IV	PD-1+A	4	SD	NDB	5.53	1	Negative(<1%)	0
Pat-240	M	70	EVER	LUAD	NGS(68 gene panel)	IV	PD-1+C	1	PR	NE	1.40	0	Negative(<1%)	0

Continued on following page

Patient_ID	Gender[1]	Age	Smoking history	Cancer_Type	NGS panel[2]	Stage	Treatment_Type[3]	lines_of_ICIs_treatment[4]	BOR[5]	DCB_status[6]	PFS(months)	PFS_status[7]	PDL1_status[8]	SMO_status[9]
Pat-241	M	34	NE	LUAD	NGS(68 gene panel)	IIIB	PD-1+C	1	PR	NDB	5.43	1	Unknown	0
Pat-242	M	63	NEVER	NSCLC	NGS(68 gene panel)	IV	PD-1	2	SD	NDB	2.97	1	Strong(>50%)	P434L(7.29%)
Pat-243	M	71	EVER	LUSC	NGS(68 gene panel)	IIIB	PD-1+C	1	PR	DCB	9.77	1	Negative(<1%)	0
Pat-244	M	64	EVER	LUSC	NGS(68 gene panel)	IV	PD-1	1	PR	DCB	20.40	0	Strong(>50%)	0
Pat-245	F	83	EVER	LUSC	NGS(68 gene panel)	IIIB	PD-1	1	SD	DCB	8.13	1	Weak(1-49%)	0
Pat-246	M	58	NE	LUSC	NGS(68 gene panel)	IV	PD-1	4	SD	DCB	13.80	0	Unknown	0
Pat-247	M	57	EVER	LUSC	NGS(68 gene panel)	IV	PD-1	1	SD	NDB	1.73	1	Unknown	0
Pat-248	M	61	EVER	LUSC	NGS(68 gene panel)	IV	PD-1	2	PR	DCB	16.57	1	Weak(1-49%)	0
Pat-249	M	67	EVER	NSCLC	NGS(68 gene panel)	IV	PD-1+C	2	PD	NDB	0.87	1	Strong(>50%)	0
Pat-250	M	60	EVER	LUSC	NGS(68 gene panel)	IV	PD-1	2	PD	NDB	1.47	1	Unknown	0
Pat-251	M	59	EVER	LUSC	NGS(68 gene panel)	IV	PD-1	1	SD	DCB	17.33	1	Negative(<1%)	0
Pat-252	M	45	EVER	LUSC	NGS(68 gene panel)	IV	PD-1	3	SD	DCB	6.90	1	Strong(>50%)	0
Pat-253	M	63	EVER	LUSC	NGS(68 gene panel)	IIIB	PD-1	2	PD	NDB	0.93	1	Unknown	0
Pat-254	M	67	NEVER	LUSC	NGS(68 gene panel)	IV	PD-1	4	SD	DCB	15.17	1	Negative(<1%)	0
Pat-255	M	66	EVER	LUSC	NGS(68 gene panel)	IV	PD-1+C	1	PR	DCB	27.67	0	Unknown	0
Pat-256	M	63	EVER	LUSC	NGS(68 gene panel)	IIIB	PD-1	2	PD	NDB	1.33	1	Negative(<1%)	0
Pat-257	F	63	NEVER	LUSC	NGS(68 gene panel)	IV	PD-1+C	1	PR	DCB	9.83	1	Strong(>50%)	0
Pat-258	M	72	EVER	LUSC	NGS(68 gene panel)	IV	PD-1	2	PD	NDB	3.23	1	Weak(1-49%)	0
Pat-259	M	69	EVER	LUSC	NGS(68 gene panel)	IV	PD-1	1	SD	DCB	8.60	0	Negative(<1%)	0
Pat-260	M	54	EVER	LUSC	NGS(68 gene panel)	IV	PD-1+C	1	PD	NDB	0.57	1	Weak(1-49%)	0
Pat-261	M	64	EVER	LUSC	NGS(68 gene panel)	IIIB	PD-1	2	SD	NDB	5.50	1	Negative(<1%)	0
Pat-262	M	62	NEVER	LUSC	NGS(68 gene panel)	IIIB	PD-1+C	1	SD	NDB	5.93	1	Unknown	0
Pat-263	M	65	EVER	LUSC	NGS(68 gene panel)	IIIB	PD-1+C	1	PR	DCB	14.17	1	Weak(1-49%)	0
Pat-264	M	55	EVER	LUSC	NGS(68 gene panel)	IIIB	PD-1	3	PD	NDB	0.70	1	Strong(>50%)	0
Pat-265	M	68	EVER	LUSC	NGS(68 gene panel)	IV	PD-1+C	2	SD	DCB	7.80	1	Weak(1-49%)	0
Pat-266	M	55	EVER	LUSC	NGS(68 gene panel)	IIIB	PD-1+C	2	SD	DCB	13.37	1	Negative(<1%)	0
Pat-267	M	64	EVER	LUSC	NGS(68 gene panel)	IIIB	PD-1	2	PD	NDB	0.90	1	Negative(<1%)	0
Pat-268	M	60	NEVER	LUSC	NGS(68 gene panel)	IV	PD-1+A	3	SD	NDB	3.40	1	Unknown	0
Pat-269	M	66	NEVER	LUSC	NGS(68 gene panel)	IV	PD-1	1	PD	NDB	1.33	1	Weak(1-49%)	0
Pat-270	M	48	NEVER	LUSC	NGS(68 gene panel)	IIIB	PD-1	2	SD	NDB	5.60	1	Negative(<1%)	0
Pat-271	M	65	NEVER	LUSC	NGS(68 gene panel)	IIIB	PD-1	2	SD	DCB	15.37	0	Unknown	M781V (37.69%)
Pat-272	M	62	EVER	LUSC	NGS(68 gene panel)	IIIB	PD-1+C	1	PR	DCB	11.93	1	Negative(<1%)	0
Pat-273	M	70	EVER	LUSC	NGS(68 gene panel)	IIIB	PD-1	2	PR	DCB	8.67	1	Negative(<1%)	S590T(48.6%)
Pat-274	M	67	EVER	LUSC	NGS(68 gene panel)	IIIB	PD-1+C	1	PR	DCB	15.23	1	Weak(1-49%)	0
Pat-275	M	63	NEVER	LUSC	NGS(68 gene panel)	IV	PD-1+C	2	PD	NDB	0.53	1	Weak(1-49%)	0
Pat-276	M	62	NEVER	LUSC	NGS(68 gene panel)	IV	PD-1+C	1	PR	DCB	6.70	0	Negative(<1%)	0
Pat-277	M	60	EVER	LUSC	NGS(68 gene panel)	IIIB	PD-1	4	PD	DCB	10.37	1	Weak(1-49%)	0
Pat-278	M	58	EVER	LUSC	NGS(68 gene panel)	IIIB	PD-1+C	2	SD	NDB	3.60	1	Weak(1-49%)	0
Pat-279	M	52	EVER	NSCLC	NGS(68 gene panel)	IV	PD-1	1	PD	NDB	0.67	1	Strong(>50%)	0
Pat-280	M	66	EVER	NSCLC	NGS(68 gene panel)	IV	PD-1+C	1	SD	NDB	4.87	1	Negative(<1%)	0
Pat-281	M	65	EVER	LUSC	NGS(68 gene panel)	IV	PD-1	1	PR	DCB	11.33	1	Strong(>50%)	0
Pat-282	M	68	NEVER	LUSC	NGS(68 gene panel)	IV	PD-1	3	SD	DCB	8.07	1	Negative(<1%)	0
Pat-283	M	66	EVER	LUSC	NGS(68 gene panel)	IV	PD-1+C	1	SD	DCB	9.10	0	Weak(1-49%)	0
Pat-284	M	66	NEVER	LUSC	NGS(68 gene panel)	IIIB	PD-1+C	1	SD	DCB	6.60	0	Weak(1-49%)	0
Pat-285	M	63	EVER	NSCLC	NGS(68 gene panel)	IV	PD-1+C	1	PD	NDB	2.20	1	Weak(1-49%)	0
Pat-286	M	58	EVER	LUSC	NGS(68 gene panel)	IV	PD-1+C	1	PR	NDB	4.20	1	Unknown	A540V(29.31%)
Pat-287	M	63	NEVER	NSCLC	NGS(68 gene panel)	IV	PD-1+C	1	PR	NDB	5.90	1	Negative(<1%)	0
Pat-288	M	50	EVER	LUSC	NGS(68 gene panel)	IV	PD-1	1	PR	DCB	9.80	0	Unknown	0
Pat-289	M	64	EVER	NSCLC	NGS(68 gene panel)	IIIB	PD-1+C	1	PR	DCB	8.60	0	Strong(>50%)	G355S(25.08%)
Pat-290	M	62	NE	LUSC	NGS(68 gene panel)	IV	PD-1+C	2	PR	NDB	3.40	1	Negative(<1%)	0
Pat-291	M	69	EVER	LUSC	NGS(68 gene panel)	IV	PD-1	2	PD	NDB	0.63	1	Strong(>50%)	0
Pat-292	M	57	EVER	LUSC	NGS(68 gene panel)	IV	PD-1+C	1	SD	NE	3.50	0	Negative(<1%)	0
Pat-293	M	75	NEVER	LUSC	NGS(68 gene panel)	IV	PD-1+C	1	SD	NE	2.40	0	Weak(1-49%)	0
Pat-294	M	81	EVER	LUSC	NGS(68 gene panel)	IIIB	PD-1	1	SD	NE	2.80	0	Unknown	0
Pat-295	M	80	EVER	LUSC	NGS(68 gene panel)	IIIB	PD-1+C	1	PR	NE	3.30	0	Weak(1-49%)	0
Pat-296	M	77	EVER	LUSC	NGS(68 gene panel)	IV	PD-1+C	1	PR	NE	2.57	0	Negative(<1%)	0
Pat-297	M	69	EVER	LUSC	NGS(68 gene panel)	IIIB	PD-1	2	SD	NDB	4.03	1	Negative(<1%)	0
Pat-298	M	64	EVER	LUSC	NGS(68 gene panel)	IIIB	PD-1+C	1	SD	NE	3.80	0	Negative(<1%)	0
Pat-299	M	69	NEVER	LUSC	NGS(68 gene panel)	IV	PD-1+C	1	SD	NDB	2.77	1	Weak(1-49%)	0
Pat-300	M	64	EVER	LUSC	NGS(68 gene panel)	IIIB	PD-1+C	1	SD	NDB	3.70	1	Strong(>50%)	0
Pat-301	M	56	NEVER	LUSC	NGS(68 gene panel)	IV	PD-1+A	2	SD	NE	3.80	0	Weak(1-49%)	0
Pat-302	M	72	EVER	LUSC	NGS(68 gene panel)	IV	PD-1+C	2	PD	NDB	1.03	1	Negative(<1%)	0
Pat-303	M	66	EVER	NSCLC	NGS(68 gene panel)	IV	PD-1+C	1	PR	DCB	6.67	1	Weak(1-49%)	0
Pat-304	M	56	EVER	LUSC	NGS(68 gene panel)	IIIB	PD-1+C	1	PR	NE	3.13	0	Unknown	0
Pat-305	M	47	EVER	LUSC	NGS(68 gene panel)	IV	PD-1+C	4	PD	NDB	1.67	1	Negative(<1%)	0
Pat-306	M	64	NEVER	LUSC	NGS(68 gene panel)	IV	PD-1	1	SD	NE	2.30	0	Strong(>50%)	0
Pat-307	M	61	EVER	NSCLC	NGS(68 gene panel)	IIIB	PD-1+C	1	PR	NE	3.30	0	Weak(1-49%)	0
Pat-308	M	69	EVER	LUSC	NGS(68 gene panel)	IV	PD-1	1	PD	NDB	0.97	1	Weak(1-49%)	0
Pat-309	M	79	EVER	LUSC	NGS(68 gene panel)	IIIB	PD-1	1	PR	NE	2.13	0	Strong(>50%)	0
Pat-310	M	50	NEVER	LUSC	NGS(68 gene panel)	IIIB	PD-1	1	SD	NE	1.40	0	Unknown	0
Pat-311	M	66	NEVER	LUSC	NGS(68 gene panel)	IV	PD-1+C	1	PR	NE	1.77	0	Negative(<1%)	0
Pat-312	M	65	EVER	LUSC	NGS(68 gene panel)	IIIB	PD-1+C	1	PD	NDB	1.47	1	Weak(1-49%)	0
Pat-313	M	62	EVER	NSCLC	NGS(68 gene panel)	IV	PD-1+C	2	SD	NE	0.73	0	Negative(<1%)	0
Pat-314	M	64	EVER	LUSC	NGS(68 gene panel)	IV	PD-1	2	NE	NE	0.83	0	Unknown	0

Gender[1] F: Femal patients, M: Male patients.

NGS panel[2]: Identified by NGS according to Lung Core Panel with 68 lung cancer-related genes.

Treatment_Type[3]: PD-1: PD-1 inhitotor, PD-1+A: combination of PD-1 inhitotor and anti-vascular therapy, PD-1+C: combination of PD-1 inhitotor and chemotherapy.

lines_of_ICIs_treatment[4]: 1: 1st line ICIs treatment, 2: ＞1st line ICIs treatment.

BOR[5]: Response Evaluation Criteria in Solid Tumors (RECIST) version 1.1.

DCB_status[6]: DCB CR/PR/SD lasted >6months, NDB PD within 6months, NE CR/PR/SD <6months.

PFS _status[7]: Event (1) or censor (0) for PFS.

PDL1_status[8]: Strong(PD-L1 pos>50%), Weak(PD-L1 pos 1-49%), Negative(PD-L1 pos <1%), Unknown no PD-L1 record.

SMO_status[9]: SMO mutation, 0 SMO wildtype.
